# Supplementary material for: Integrating a brief mental health intervention into primary care services for patients with HIV and diabetes in South Africa: study protocol for a trial-based economic evaluation
Source: BMJ Open. 2019 May 14;9(5):e026973. doi: 10.1136/bmjopen-2018-026973 (PMC6530312; doi:10.1136/bmjopen-2018-026973)
Supplement: Author's manuscript [file bmjopen-2018-026973.draft_revisions.pdf]

BMJ Open

BMJ Open is committed to open peer review. As part of this commitment we make the peer review history of every article we publish publicly available.

When an article is published we post the peer reviewers' comments and the authors' responses online. We also post the versions of the paper that were used during peer review. These are the versions that the peer review comments apply to.

The versions of the paper that follow are the versions that were submitted during the peer review process. They are not the versions of record or the final published versions. They should not be cited or distributed as the published version of this manuscript.

BMJ Open is an open access journal and the full, final, typeset and author-corrected version of record of the manuscript is available on our site with no access controls, subscription charges or pay-per-view fees (http://bmjopen.bmj.com).

If you have any questions on BMJ Open's open peer review process please email info.bmjopen@bmj.com

BMJ Open

# Integrating a Brief Mental Health Intervention into Primary Care Services for HIV and Diabetes Patients in South Africa: Study Protocol for a Trial Based Economic Evaluation.

| | |
|---|---|
| Journal: | *BMJ Open* |
| Manuscript ID | bmjopen-2018-026973 |
| Article Type: | Protocol |
| Date Submitted by the Author: | 28-Sep-2018 |
| Complete List of Authors: | Mafunda, Vimbayi; University of Cape Town School of Public Health and Family Medicine, Health Economics Unit;<br>Myers, Bronwyn; South African Medical Research Council, Alcohol and Drug Abuse Research Unit<br>Sorsdahl, Katherine<br>Lund, Crick; University of Cape Town, Alan J Flisher Centre for Public Mental Health<br>Naledi, Tracey; University of Cape Town<br>Cleary, S; University of Cape Town, Health Economics Unit |
| Keywords: | cost-effectiveness analysis, common mental disorders, HIV, Diabetes, integrated treatment |
| | |

SCHOLARONE™
Manuscripts

# Integrating a Brief Mental Health Intervention into Primary Care Services for HIV and Diabetes Patients in South Africa: Study Protocol for a Trial Based Economic Evaluation.

**Authors:** Vimbayi Mutyambizi-Mafunda[1], Bronwyn Myers[2,3], Katherine Sorsdahl[4], Crick Lund[4,5], Tracey Naledi[6], Susan Cleary[1]

[1] Health Economics Unit, School of Public Health and Family Medicine, University of Cape Town, Cape Town, South Africa.

[2] Alcohol, Tobacco and Other Drug Research Unit, South African Medical Research Council, Francie van Zyl Drive, Tygerberg, Cape Town, 7505, South Africa

[3] Division of Addiction Psychiatry, Psychiatry and Mental Health, University of Cape Town, Cape Town, South Africa.

[4]Alan J Flisher Centre for Public Mental Health, Department of Psychiatry and Mental Health, University of Cape Town, Cape Town, South Africa.

[5] Centre for Global Mental Health, Institute of Psychiatry, Psychology and Neuroscience, King's College, London, UK.

[6] Western Cape Department of Health, 8 Riebeeck Street, Cape Town, South Africa.

**Author Email addresses:**

Vimbayi Mutyambizi-Mafunda - vimbayi.mafunda@uct.ac.za

Bronwyn Myers – Bronwyn.Myers@mrc.ac.za

Katherine Sorsdahl – katherine.sorsdahl@uct.ac.za

Crick Lund – crick.lund@uct.ac.za

Tracey Naledi – Tracey.Naledi@hiv-research.org.za

Susan Cleary – susan.cleary@uct.ac.za

**Corresponding Author**

**Name:** Vimbayi Mutyambizi-Mafunda

**Postal Address:** Health Economics Unit, University of Cape Town, School of Public Health & Family Medicine, Health Sciences Faculty, Anzio Road, Observatory, Cape Town,7925, South Africa.

**Email address:** vimbayi.mafunda@uct.ac.za

**Telephone Number:** +27 21 406 6558

**Word count:** 4038

For peer review only

## ABSTRACT

**Introduction:** Depression and alcohol use disorders are international public health priorities for which there is a substantial treatment gap. Brief mental health interventions delivered by lay health workers in primary care services may reduce this gap. There is limited economic evidence assessing the cost-effectiveness of such interventions in low-and middle-income countries (LMIC). This paper describes the proposed economic evaluation of a health systems intervention testing the effectiveness and cost-effectiveness of two task-sharing approaches to integrating services for common mental disorders with HIV and Diabetes primary care services.

**Methods and analysis:** This evaluation will be conducted as part of a three-armed cluster-randomised controlled trial of clinical effectiveness. Trial clinical outcome measures will include primary outcomes for risk of depression and alcohol use, and secondary outcomes for risk of chronic disease (HIV and Diabetes) treatment failure. The cost effectiveness analysis will evaluate cost per unit change in AUDIT and CESD scores as well as cost per unit change in HIV RNA viral load and HBA1c, producing results of provider and patient cost per patient year for each study arm and chronic disease. The cost utility analyses will provide results of cost per quality adjusted life year (QALY) gained. Additional analyses relevant for implementation including budget impact analyses will be conducted to inform the development of a business case for scaling up the country's investment in mental health services.

**Ethics and dissemination:** The Western Cape Department of Health (WCDoH) (WC2016_RP6_9), the South African Medical Research Council (EC 004-2/2015), the University of Cape Town (089/2015), and Oxford University (OxTREC 2-17) provided ethical approval for this study.

**Trial registration**: Pan African Clinical Trials registry (registration number PACTR201610001825405).


**Keywords:** cost-effectiveness analysis, common mental disorders, HIV, Diabetes, integrated treatment

## ARTICLE SUMMARY

### Strengths and limitations of this study

- This study will provide some of the first empirical evidence of the cost-effectiveness of integrating services for common mental disorders in the primary care offering for HIV and Diabetes.
- The study will generate findings that may guide priority setting through application of QALY based outcomes and decision-making around the implementation of mental health counselling within the health system.
- This paper adds to the growing body of protocols for economic evaluations conducted alongside RCTs.
- As health service use is self-reported, there is a possibility for recall bias, to limit this, a one month recall period will be used for ambulatory services and a 6 month recall for hospitalisation, and hospitalisations will be validated through the Department of Health Data Centre.
- The one year follow up period may reflect outcomes that do not fully represent the full benefit that patients and the health system could gain through the availability of this service; further work using an extended follow-up period may be proposed.



For peer review only - http://bmjopen.bmj.com/site/about/guidelines.xhtml

## INTRODUCTION

Common mental disorders (CMDs) including depression, anxiety and alcohol use disorders are highly prevalent conditions that impose a significant societal cost and impact on quality of life[1]. Globally, these conditions have been shown to be the leading cause of non-fatal burden of disease[2]. The disabling impact of these conditions is particularly significant in patients suffering from chronic physical conditions[3]. CMD co-morbidity has been shown to: exacerbate major modifiable risk factors for chronic disease; contribute to chronic disease progression; increase the prevalence of preventable complications and disability; result in poor adherence; and ultimately lead to treatment failure[1], [4], [5]. There is a plethora of evidence on the impact of depression [5], [6]and alcohol use disorders (AUD)[7], [8] on diabetes outcomes. Likewise, alcohol consumption[9], [10] and depressive conditions[11], [12] have been associated with poor HIV outcomes. In addition high-income country (HIC) evidence suggests that patients with these multi-morbidities have higher health care costs even after adjusting for the costs of mental health care [13]; suggesting potential cost savings from investing in mental health treatment for patients with multi-morbidities[14].

There is a pressing need for evidence on cost-effective strategies for integrating mental health care into primary health services, particularly in low-and middle-income countries (LMICs) where health systems are typically fragile and chronically underfunded. In these health systems, an investment in mental health services at primary care level for chronic disease patients has the potential to result in significant returns on investment due to reduced chronic disease treatment costs. South Africa - with the world's largest ART programme[15], an increasing prevalence of diabetes[16] and a huge unmet need for CMD treatment[17] - is one LMIC that may benefit from this integrated approach.

Task-sharing [18], [19] involves re-distributing tasks from specialist providers to non-specialist health workers [20] with specialists providing on-going support to these health workers. Task-sharing is a common strategy for the provision of primary care services in constrained LMIC settings[21],[22],[24]. Due to the long lead times in training mental health staff and financial resource limitations, the task-sharing discourse has found appeal in the policy discussions around introducing and expanding mental health care in LMICs[22],[23],[24]. These discussions are centred on using task-shared approaches[25] to upskill lay health workers to integrate mental health services such as brief counselling interventions into primary care[26],[27],[28]. Task-sharing the psychosocial counselling of patients who have chronic co-morbidities to lay health workers is a particularly appealing and potentially cost-effective strategy to treat patients and support their adherence to chronic medication[29],[30] and therefore limit overall health system costs of care[5],[31].

While task-sharing the treatment for CMDs from mental health professionals to lower level health care workers has been suggested as an effective way to deliver services within the context of scarce human resources [5] and has been shown to be cost effective in some HIC settings, there is little if any economic evidence to support the implementation of such strategies in LMICs. This lack of evidence limits decision making around the allocation of resources for these services. Economic evidence on the institutional investment, cost-effectiveness and budget impact of this approach is necessary for empirically-informed health service planning. This work presents a protocol for an economic evaluation of a task-sharing strategy for delivering a brief mental health counselling intervention to patients with comorbid HIV or Diabetes in South Africa.



For peer review only - http://bmjopen.bmj.com/site/about/guidelines.xhtml

**METHODS AND ANALYSIS**

**Trial design**

This study is nested in Project MIND, a cluster randomised control trial across 24 public primary care facilities [32]. Myers et al., (2018) describes the trial in detail [32].The trial will compare the effectiveness of treatment as usual (MIND_TAU) and two alternative methods for integrating a psychosocial intervention into the primary care services for HIV and Diabetes. The two models will be the dedicated and designated models of care. In the dedicated approach (MIND_DED), community health workers (CHWs) will be hired and added to the facility staff complement and will dedicate their time to delivering the new counselling service. In the designated approach (MIND_DES CHWs already working in the facility will be designated to deliver the service in addition to their other chronic disease-related activities such as adherence counselling for HIV and health promotion. Supervision and de-briefing of all the counsellors in the active arms will be task-shifted to registered psychological counsellors in line with the national mental health policy framework [33]. The Western Cape Department of Health (WCDoH) (WC2016_RP6_9), the South African Medical Research Council (EC 004-2/2015), the University of Cape Town (089/2015), and Oxford University (OxTREC 2-17) approved the study.

***Intervention and comparator***

The counselling programme delivered in MIND-DED and MIND-DES is based on Motivational Interviewing (MI) and Problem Solving Therapy (PST)[34]. It is delivered in three sessions over six weeks with one optional booster session. The MI component provides psychoeducation about depression and hazardous alcohol use in relation to chronic disease, builds readiness for counselling and helps participants develop goals for counselling. The PST component targets maladaptive coping strategies and builds skills and strategies for dealing with life problems. Patients will receive individual face-to-face counselling from CHWs and take-home booklets to support self-learning between sessions. Registered psychological counsellors, supervised by psychologists, will train the counsellors (see [32] for more detail). Treatment as usual will include providing the patient with a resource guide containing information around the mental health and substance abuse services available within their health sub-district, and a referral to a service provider of their choice.

***Outcome Measures***

The primary outcomes for the trial are measures of psychosocial functioning, specifically changes in self-reported risk of depression as measured by CES-D (Centre for Epidemiological Studies scale on Depression)[35] and self-reported hazardous alcohol use as measured by the AUDIT (Alcohol Use Disorder Identification Test) [36]. Secondary outcomes include biological measures aimed at assessing changes in chronic disease treatment response, specifically HbA1c for diabetes and HIV-1 RNA viral loads for HIV. Other secondary outcome measures include self-reported adherence to chronic disease medication and health-related quality of life measured using the EQ-5D [37]. All outcomes are measured at baseline, and at 6 and 12 month follow-up assessments (see **Figure 1)**.



For peer review only - http://bmjopen.bmj.com/site/about/guidelines.xhtml

| *Analysis* | ***Assessment/ Economic Evaluation Outcomes*** | **Measurement instrument OR Clinical Parameter** | **Assessment Timing** Baseline (Intervention start) | 6 months | 1 Year (Intervention assessment end) |
|---|---|---|---|---|---|
| | ***1º Outcomes*** | | | | |
| **CEA** | Harmful alcohol use (Self-report interview) | AUDIT | ✓ | ✓ | ✓ |
| | Depression (Self-report interview) | CES-D | ✓ | ✓ | ✓ |
| | ***2º Outcomes*** | | | | |
| | Diabetes treatment failure risk | HbA1c | ✓ | ✓ | ✓ |
| | | BMI | | | |
| | HIV treatment failure risk | HIV-1 RNA (HIV-viral load) | ✓ | ✓ | ✓ |
| | Chronic Disease Medication Adherence | Visual analogue Scale and CASE adherence Index | ✓ | ✓ | ✓ |
| **CUA** | ***Multi-attribute Outcomes*** | | | | |
| | QALY | EQ-5D | ✓ | ✓ | ✓ |

**Figure 1** Economic Evaluation: Analyses, Outcomes, Measurement and Assessment Timing

***Sample Size and Patient Population***

Recruitment will be restricted to consenting adult patients who meet criteria for hazardous alcohol use or depression using the AUDIT or CES-D. These patients are already diagnosed and receiving treatment for diabetes or HIV, but not currently receiving treatment for a CMD. The trial requires a sample size of 1200 patients (600 with HIV and 600 with diabetes) to detect reductions in hazardous alcohol use and risk of depression at 12 month follow-up. The sample size calculations are based on a cluster randomised design with two active arms and a control arm. This clinical trial is powered for clinical rather than economic outcomes[38].

**MIND Economic Evaluation**

**Objective**

The objective of this prospective health systems economic evaluation is to estimate for alternative intervention options the:

i. Full economic costs of integrating the mental health intervention into primary care;
ii. Cost-effectiveness in terms of the incremental cost per unit of improvement in disease specific outcome (CES-D, AUDIT, HIV-1 RNA, HBA1c);
iii. Cost-utility in terms of the incremental cost per Quality Adjusted Life Year (QALY) gained and;
iv. Budget impact of scaling up the intervention.

To achieve these objectives, the incremental cost-effectiveness ratio (ICER) will be calculated by ordering the MIND_DED, the MIND_DES and MIND_TAU alternatives from least cost to highest cost and calculating the difference in costs and outcomes against the next less costly, non-dominated alternative or against the common baseline of MIND_TAU.



For peer review only - http://bmjopen.bmj.com/site/about/guidelines.xhtml

**Perspective**
The aim of the economic evaluation is to inform health service decision makers about the costs, outcomes, economic efficiencies and budget impact of introducing task-shared psychosocial counselling to their chronic disease patient body in a resource-scarce context. As such, the health system economic evaluation will be conducted from a provider perspective, reflecting health service budget constraints. In line with good practise recommendations, the analyses will also be presented from a societal perspective[39].

**Time Horizon and Discount Rate**
As the health consequences of the intervention will be evaluated over a period extending longer than one year, a discount rate will be applied to reflect differentials in timing[40]. A three percent discount rate will be used for discounting both costs and outcomes to allow comparability with other cost-effectiveness analyses[41].

**Estimating intervention effects**
Single study-based estimates of clinical effectiveness will be obtained from the trial, which will provide the primary and secondary outcome measures previously described. In addition to the disease-specific primary outcomes, and the secondary HIV and diabetes outcomes which will be applied in the cost-effectiveness analysis, the QALY will also be calculated as an end-point that will be used in the cost-utility analysis. The ICER resulting when using a QALY measure represents the additional cost associated with accruing another QALY as one compares strategies (that is, the incremental cost per QALY gained). The QALY allows comparison of ICERs across different diseases and interventions[41]. QALYs are calculated by multiplying the length of time in a health state by the health-related quality of life (HRQoL) of the health state. The EQ-5D is the most widely used instrument for measuring HRQoL for cost-utility analyses [42]. In South Africa it has been validated and translated into Afrikaans and isiXhosa, two of the country's official languages[43]. The EQ-5D is a self-assessment of health status across five domains which include mobility, self-care, usual activities, pain and discomfort and anxiety/depression[37]. In this study, the EQ-5D-3L [44] will be used to measure HRQoL. A single summary index score will be generated using preference weights derived from a time trade-off (TTO) based valuation survey conducted in Zimbabwe in addition to the commonly used United Kingdom value set; the TTO is the most common valuation technique used in LMICs[45]. As the EQ-5D will be administered at all the assessment intervals (baseline, 6 months and 12-months post-enrolment) there will be three observations of the EQ-5D scores for each patient in the trial. The QALY gained per patient over the trial period will be calculated by measuring the area under the quality of life curve[46].

**Estimating costs**
***Measurement of resource use***
Provider costs will include all costs to be incurred in delivering the intervention and the health care providers accessed by the patient. Routine data linked to intervention protocols will be collected and used to assess resources directly consumed in the provision of the intervention, including supplies, manuals, and patient education materials. Facility observations of intervention delivery and the HIV and Diabetes service delivery will be conducted for the economist to understand the intervention and the facility context; these observations will complement intervention cost data collection. The costs of intervention focused staff training and facility-level institutional strengthening and capacity development through organisational readiness workshops [47] will be included in the cost analysis in order to capture the full economic costs of integrating the intervention into the primary care service for chronic disease patients.



For peer review only - http://bmjopen.bmj.com/site/about/guidelines.xhtml

A time and motion tool (TMT) developed for this evaluation will measure counsellor time usage in delivering the intervention for both active arms in the study. Time tools have been used in analysis of human resource requirements for expanding access to HIV[48], [49] and TB care[50] using lay health workers and have proved useful in highlighting the scope of duties of this cadre. To our knowledge there is limited evidence in the South African context on time costs of CHW-delivered mental health counselling in the South African context. The TMT will enable an analysis of differences in counsellor time usage in the different intervention modalities, indicating opportunities for scale and scope efficiencies. Although mental health nurses and drugs are specified line items in the primary health care budget treatment is still limited to upward referrals in the public health system and to NGOs and private providers. This suggests that the budgetary allocation of resources is yet to translate into a meaningful delivery of mental services in South Africa's primary care setting. It is anticipated that the costs associated with the TAU option will be minimal and any referral costs will be captured through patient self-report of services they have used.

To quantify the staff time costs for the HIV and Diabetes services, a gross costing methodology will be used where salary data available from expenditure reports will be apportioned based on the amount of time staff spend on the service. Staff time will be determined through review of facility organograms and interviews with senior managers. This top-down methodology will also be used in estimating the HIV and Diabetes medicines and diagnostics costs by applying protocol-based facility utilisation statistics. To establish patient utilisation of health services beyond their HIV and Diabetes care at the primary level, we will administer a patient questionnaire developed for the study at baseline, and at the 6 and 12 month assessments. It will capture health care utilisation and associated costs of both public and private visits for other primary care services (e.g. TB treatment), hospital services, emergency department care, NGO provided care, paramedical care, and alternative traditional and faith-based therapies. In addition, it will capture the costs of any patients that are referred to mental health providers in TAU or in the other study arms. Patients will be asked to quantify the number of these additional visits, lengths of stay in hospital, and any out-of-pocket payments for health care.

***Valuation of health sector costs***

To estimate the costs of resources used directly in the intervention as well as other health provider resources used, utilisation will be multiplied by the unit costs/prices/salaries obtained from published sources and official expenditure reports of the Department of Health and other health care funders. In this setting, provider costs are typically estimated using a combination of ingredients and step-down methods.

Capital costs will include: counsellor training costs (retraining will be required after 3 years), costs of facility room space, and furniture and equipment. The cost of facility space, and furniture and equipment will be estimated using the replacement value method[41] by applying the price index for new buildings for South Africa for space and market prices for furniture and equipment. An estimated useful life of between 5-20 years for furniture and buildings will be applied as appropriate. It is recommended that a relevant discount rate of between 0-19%, reflecting the contextual conditions of the country/region in which the study is undertaken be applied when calculating a discount factor for amortising capital costs[41], therefore the capital cost estimates will be annuitized using an 8% discount rate reflecting the rate of return on government bonds in South Africa.

Direct provider costs will be allocated to the patients based on their utilisation in the different arms of the intervention. A best practise approach for estimating overheads involves using aggregated facility level costs available from global expenditure reports and apportioning these based on utilisation headcounts[51].



For peer review only - http://bmjopen.bmj.com/site/about/guidelines.xhtml

***Valuation of patient costs***

The estimation of patient costs will involve multiplying the utilisation of each service (that is the number of visits or inpatient days sourced from the patient questionnaire) by the unit costs/prices sourced from official expenditure reports and published data. Travel costs will be estimated using the cost of travel as reported by patients. Subsistence and accommodation will also be estimated by reported values from these questionnaires. Travel, subsistence and accommodation costs will relate to both the patient and their caregiver, if the patient is accompanied by a caregiver to any of these services.

***Valuation of productivity/time costs***

Losses in productivity and time spent receiving medical care will be estimated using patient-reported income losses and valuation of productive time lost using an equality of wages approach[52], [53]. We will apply this method as public health services in South Africa are typically accessed by those who have a lower socio-economic status. Using this technique, the time costs for all patients are valued at the average income reported by the survey population[52]. This approach is expected to provide a relatively accurate reflection of the opportunity cost of unpaid labour and is favoured over the minimum wage approach in this context as a large proportion of the patients are unemployed resulting in a possible overestimate of the value of the time loss if the minimum wage is used[52],[54].

Resources used will be valued at 2018/2019 prices and to allow comparability with other studies will be converted from South African Rand (ZAR) to United States Dollars (US$), using appropriate conversion rates for the same period obtained from OANDA, an online currency exchange rate conversion site. Costs incurred during earlier periods (such as manual development) will be inflated using the Consumer Price Index to reflect 2018/2019 prices. The measurement and valuation of intervention and health services costs are summarized in **table 1**.



For peer review only - http://bmjopen.bmj.com/site/about/guidelines.xhtml

| IDENTIFICATION | MEASUREMENT | | VALUATION |
|---|---|---|---|
| **Cost component** | **Resources listed** | **Quantities of each** | **Valuation** |
| ***INTERVENTION*** | | | |
| *Capital costs* | | | |
| Facility Readiness Workshops | Number of staff | Time from trial data | Annual salary X proportion of time |
| Intervention training | Number of staff | Time from trial data | Annual salary X proportion of time |
| Counselling room | Space | Square meters | Current replacement value (CRV) X estimated useful life (EUL) |
| Furniture & equipment | e.g. Tape recorders | Inventory | CRV X EUL |
| Vehicles | Vehicles | Inventory | CRV X EUL |
| *Recurrent costs* | | | |
| Counselling personnel | Number of staff (Lay counsellors; registered counsellors; clinical psychologist) | Time from time and motion tool and trial records | Annual salary X proportion of time |
| Counselling supplies | Manuals, notebooks, pens | Number used - from trial data | Market value |
| Non-intervention personnel | Number of staff | Time allocation | Annual salary X proportion of time |
| Utilities | Electricity, water, other utilities, phone, cleaning, transport, and stationery | Routine facility utilisation | Routine facility expenditure data |
| ***HIV/DIABETES SERVICE*** | | | |
| *Capital costs* | | | |
| Buildings | Space used for HIV/Diabetes service | Measure space used & total building space | Current replacement value, split equally across HIV/Diabetes patient headcount |
| Equipment& furniture | Equipment and furniture used for HIV/Diabetes service | Inventory | Current replacement value, split equally across HIV/Diabetes patient headcount |
| Vehicles | Vehicles used for HIV/Diabetes service | Inventory | Current replacement value, split equally across HIV/Diabetes patient headcount |
| *Recurrent costs* | | | |
| HIV/Diabetes personnel | Number of staff and staff time spent on HIV/Diabetes service | Time spent on HIV/Diabetes service based on staff establishment | Salary information divided by the time spent on HIV/Diabetes service split equally across HIV/Diabetes patient headcount |
| Drugs | ARV/Diabetes medication | Average utilisation based on treatment protocol | Cost of ARV/Diabetes medication based on national tender prices |
| Diagnostic tests | CD4 test, diabetes monitoring tests etc. | Average utilisation based on treatment protocol | Costing of diagnostic testing based on National Health Laboratory Service (NHLS) prices |
| Non-HIV/Diabetes personnel | Managerial, cleaning and security staff | Routine facility utilisation | Routine facility expenditure data split equally across all patient headcounts |
| Building operating and maintenance | Electricity, water, other utilities, phone, cleaning, transport, and stationery | Routine facility utilisation | Routine facility expenditure data split equally across all patient headcounts |
| ***OTHER RELATED HEALTH CARE PROVIDERS*** | | | |
| Public clinic visit | Average cost per visit | Patient baseline interview | Department of Health Expenditure reports |
| Public hospital ER/OPD visit | Average cost per OPD visit | Patient baseline Interview | Department of Health Expenditure reports |
| Public hospital Inpatient visit | Average cost per IPD visit | Patient baseline Interview | Department of Health Expenditure reports |
| Private hospital ER/OPD visit | Average cost per OPD visit | Patient baseline Interview | Board of Health Care Funders/Council of medical Scheme Annual Reports |
| Private doctor consultation | Average consultation cost | Patient baseline Interview | Board of Health Care Funders/Council of medical Scheme Annual Reports |
| Traditional healer visit | Average cost of a visit | Patient baseline Interview | Secondary data sources (literature) |
| Faith based healer visit e.g. church | Average cost of a visit | Patient baseline Interview | Secondary data sources (literature) |
| NGO/Support group | Average cost of a visit | Patient baseline Interview | Department of Health Expenditure reports |

**Table 1:** Identifying, measuring and valuing intervention and health services costs



For peer review only - http://bmjopen.bmj.com/site/about/guidelines.xhtml

## Analyses

### *Statistical analysis of costs and effects*

Mean total costs, intervention costs, health services costs, patient costs, and productivity/time costs will be calculated. Cost differences between the MIND-TAU, and the MIND-DES and MIND-DED alternatives will be calculated. Sampling uncertainty for cost data will be estimated using statistical methods that account for the non-normal distribution of costs. Effect in terms of CES-D, AUDIT, HIV viral load, HBA1c, and the QALY will be analysed using linear regression on intervention type.

### *Determining cost-effectiveness*

The analysis will be conducted in line with the CHEERS (Consolidated Health Economic Evaluation Reporting Standards) guidelines[55].Decision analytical models[56] integrating the cost and outcomes of the different intervention alternatives will be developed to assess cost-effectiveness. As a meaningful impact of the MIND-DES and MIND-DED on both costs and effects is anticipated, ICERs will be estimated in terms of incremental cost per natural unit and/or intermediate outcome for the cost effectiveness analysis (CEA), and incremental cost per QALY gained for the cost utility analysis (CUA). As the trial is focused on health systems strengthening for the delivery of mental health services, the decision rule applies to maximising health within the health care budget constraint. Therefore ICERs will be calculated from the health services perspective. Patient costs will be collected and reported on separately. To satisfy the requirements of common guidelines[55] and to allow for some degree of comparability with other ICER results in our setting, the ICERs will also be presented for the societal perspective. The ICERs will be compared to cost-effectiveness thresholds that are applicable for the South African context[57].

### *Sensitivity analyses*

The decision analytical models will allow for univariate and multivariate sensitivity analyses to be conducted. This will enable the robustness of study findings to be tested by varying key parameters such as utilisation rates and assessing the impact on cost-effectiveness results. Standard areas of analytical focus will be considered, including testing sensitivity around the resource cost and prices, and theoretical controversies[58] for example: TTO weights[59] and discounting of outcomes [60], [61]. Further analyses to inform the economics of task-sharing will include sensitivity analysis focused on economies and diseconomies of scale and scope, using detailed data from the TMT [62]. This will include testing variations in staff hours worked, variations in staff mix, lay counsellor funding models, and infrastructure investments. The impact of intervention dosage on outcomes will also be assessed in the sensitivity analyses. Statistical methods including multiple imputation will be used to manage the effect of missing information on costs and effects[63]. These sensitivity analyses are summarised in **table 2**. Probabilistic sensitivity analysis (which allows application of confidence intervals to point estimates) will be used for sensitivity analyses of all relevant variables.

| **Standard parameters/methodological assumptions** | **Base-Case** | **Sensitivity Analysis** |
|---|---|---|
| Discount rate | 3% | Varied within standard ranges |
| QALYs: HRQoL weights | Derived from EQ-5D | EQ-5D: UK & Zimbabwe TTO |
| Unit costs | Data from published sources and official statistics for South Africa | Varied within plausible range |
| Missing data | Multiple imputation | Complete case analysis |
| **Study specific parameters/methodological assumptions** | | |
| Institutional costs | Derived from primary data | Varied within plausible range |
| Intervention dosage | Derived from RCT data | Varied within clinically accepted ranges |

**Table 2:** Planned Sensitivity Analyses



For peer review only - http://bmjopen.bmj.com/site/about/guidelines.xhtml

**DISCUSSION**

The delivery of psychotherapy using varying models of task-sharing to less specialised health workers in primary care has been shown to be cost-effective in HIC settings [64],[65],[66], across a range of mental health conditions[67], [68], but has limited empirical economic evidence in LMICs[62], [69], [70]. This study will provide evidence to address this gap. Through the cost-effectiveness analysis, the relative efficiency of each delivery mechanism will be established, enabling a discussion around which model produces the best outcomes given the available resources. As staff time is expected to be one of the main drivers of costs due to the nature of the intervention, we have developed a time tool that will allow us to directly measure staff time usage and investigate any potential excess capacity (hypothesized to occur with the MIND-DED counsellors), and scope economies (hypothesized to occur with the MIND-DES counsellors) which would impact the comparative efficiency of the different alternatives and thus the cost effectiveness results, as suggested in a similar study [62]. The cost-utility analysis will aid health service planners in their decision making around resource allocation and may be useful for guiding priority setting decisions both within the mental health budget space and across competing health priorities. To further support policy maker decisions, the affordability of implementing the intervention will need to be assessed, this will require further analysis using estimates of numbers in need to approximate the budget impact of equitably implementing the service[71].

Policy discussions on the integration of services to provide holistic care for patients requires economic evidence to support implementation in resource-limited contexts[72], [73]. By estimating the full economic costs including the institutional costs [74],[75],[76] of integrating the mental health treatment into the primary care offering for patients with chronic physical diseases, this study will provide much needed economic evidence to support this policy narrative[77], [78]. Empirical data on the costs of upskilling and training staff to deliver the intervention and the time costs of the alternative human resourcing strategies will also aid in understanding how this cadre of staff organise their work and may highlight opportunities for efficiency gains. This is timely in the current policy window where the idea of a "facility counsellor" is being discussed, whose role may span a range of counselling duties including: adherence counselling for TB/HIV, chronic non-communicable physical diseases and mental health counselling. The integration of services is a key health systems focus[79] requiring economic evidence[80]. The integration of services at the primary care level [81] to manage communicable and non-communicable chronicity is a global challenge [80], [82], [83] and a key strategic aim of the Department of Health [84] for expanding access to mental health services and managing chronic diseases in South Africa [22],[70]. Brief interventions for depression and alcohol use disorders lack a common outcome measure for use in cost-effectiveness analysis[85] thus limiting comparison across interventions [86]. This study will provide unique cost-effectiveness evidence using the QALY as an outcome measure for such interventions in the South African context[62]. This will allow comparability of cost effectiveness results [87] and inform value for money decision making[57]. This is of particular importance for South Africa as discussions around cost-effectiveness thresholds[57] are being increasingly presented in the health policy space in the prelude to National Health Insurance. This protocol also responds to the call to publish health economics protocols alongside trial protocols to reduce publication bias of RCT results [88].

Potential challenges to this study include recall bias due to the reliance on patient-reported health service use for our estimations of service utilisation. To limit recall bias, a one month recall period will be used for ambulatory services and 6 month recall for hospitalisations[63]. Another potential limitation is that the short follow-up period of 12 months may not capture the extent of the benefit that patients and the health system could gain through the availability of this service. For example, the degree to which the socio-economic benefits and the returns to mental health associated with such interventions [89] may not be fully evident by the 12 month end-point. This is a common problem associated with trial-based economic evaluations[90].



For peer review only - http://bmjopen.bmj.com/site/about/guidelines.xhtml

**FUTURE WORK**

Further work associated with this economic evaluation includes the use of modelling techniques to estimate the long-term benefits of the intervention both at the patient and system level and applying cost-benefit techniques to strengthen the evidence base for the mental health investment case.

**CONCLUSION**

Priority setting and decisions around the scale up of an integrated comprehensive primary care service need to be informed by micro- and macro-level economic analyses of the investment requirements, impact, and equity implications of delivering key services such as counselling. It is anticipated that the cost estimates, the CEA and budget impact analysis of this intervention will be a useful contribution to priority setting and policy making during the current reconfiguration of South Africa's health system [91].It is hoped that the economic evaluation of project MIND will provide much needed evidence on the cost-effectiveness of integrating mental health services into primary care that could support a business case for investing in these services.

**FUNDING STATEMENT**

This work is supported by the joint funded initiatives of the British Medical Research Council, Wellcome Trust and DFID (MR/M014290/1). BM is supported by the South African Medical Research Council.

**COMPETING INTERESTS STATEMENT**

The authors declare that they have no competing interests.

**AUTHOR CONTRIBUTIONS**

VM-M, SC and BM made substantial contributions to the conception and design of the study. The first draft of the manuscript was written by VM-M and critically revised by all authors (VM-M, BM,KS,CL,TL ,SC) for important intellectual content. All authors read and approved the final manuscript.

**ABBREVIATIONS**

ART: Antiretroviral therapy; AUDIT: Alcohol Use Disorders Identification Test; CEA: Cost Effectiveness Analysis; CES-D: Center for Epidemiology Scale on Depression; CHW: Community health worker; CMD: Common mental disorder; CUA: Cost Utility Analysis; HIC: High Income Country; HRQoL: Health-related quality of life; LMIC: Low- and middle-income country; MI: Motivational interviewing; NCD: Non-communicable disease; PHC: Primary health care; PST: Problem- solving therapy; QALY: Quality-adjusted life year; TMT: Time and motion tool; TAU: Treatment as usual; WCDOH: Western Cape Department of Health

**ACKNOWLEDGEMENTS**

We thank all WCDOH facilities, which have agreed to participate in the trial, and our Stakeholder Advisory Group for their contributions.



For peer review only - http://bmjopen.bmj.com/site/about/guidelines.xhtml

**REFERENCES**


[1] M. Prince *et al.*, “No health without mental health,” *Lancet*, vol. 370, no. 9590, pp. 859–877, 2007.

[2] H. a Whiteford *et al.*, “Global burden of disease attributable to illicit drug use and dependence: findings from the Global Burden of Disease Study 2010,” *Lancet*, vol. 382, no. 13, pp. 1564–1574, 2013.

[3] S. Moussavi, S. Chatterji, E. Verdes, A. Tandon, V. Patel, and B. Ustun, “Depression , chronic diseases , and decrements in health :,” *Lancet*, pp. 851–858, 2007.

[4] World Health Organisation, “Investing in Mental Health,” 2003.

[5] V. K. Ngo *et al.*, “Grand Challenges: Integrating Mental Health Care into the Non-Communicable Disease Agenda,” *PLoS Med.*, vol. 10, no. 5, pp. 1–6, 2013.

[6] L. E. Egede and C. Ellis, “Diabetes and depression: Global perspectives,” *Diabetes Res. Clin. Pract.*, vol. 87, no. 3, pp. 302–312, 2010.

[7] N. V Emanuele, T. F. Swade, and M. a Emanuele, “Consequences of alcohol use in diabetics.,” *Alcohol Health Res. World*, vol. 22, no. 3, pp. 211–219, 1998.

[8] A. T. Ahmed, A. J. Karter, and J. Liu, “Alcohol consumption is inversely associated with adherence to diabetes self-care behaviours,” *Diabet. Med.*, vol. 23, no. 7, pp. 795–802, 2006.

[9] M. G. Neuman, M. Schneider, R. M. Nanau, and C. Parry, “Alcohol consumption, progression of disease and other comorbidities, and responses to antiretroviral medication in people living with HIV,” *AIDS Res. Treat.*, vol. 2012, 2012.

[10] M. Schneider, M. Neuman, M. Chersich, and C. Parry, “Alcohol and antiretroviral therapy - A lethal cocktail,” *J. AIDS Clin. Res.*, vol. 3, pp. 1–8, 2012.

[11] J. a. Cook *et al.*, “Depressive symptoms and AIDS-related mortality among a multisite cohort of HIV-positive women,” *Am. J. Public Health*, vol. 94, no. 7, pp. 1133–1140, 2004.

[12] A. Ammassari, “Correlates and predictors of adherence to highly active antiretroviral therapy: overview of published literature,” *J. Acquir. Immune Defic. Syndr.*, vol. s1, pp. s123–s127, 2002.

[13] L. E. Egede, D. Zheng, and K. Simpson, “Comorbid depression is associated with increased health care use and expenditures in individuals with diabetes,” *Diabetes Care*, vol. 25, no. 3, pp. 464–470, 2002.

[14] W. J. Katon, J. E. Russo, M. Von Korff, E. H. B. Lin, E. Ludman, and P. S. Ciechanowski, “Long-term effects on medical costs of improving depression outcomes in patients with depression and diabetes,” *Diabetes Care*, vol. 31, no. 6, pp. 1155–1159, 2008.

[15] B. M. Mayosi, J. E. Lawn, a Van Niekerk, D. Bradshaw, S. S. Abdool Karim, and H. M. Coovadia, “Health in South Africa: Changes and challenges since 2009,” *Lancet*, vol. 380, pp. 2029–2043, 2012.

[16] D. Bradshaw, K. Steyn, N. Levitt, and B. Nojilana, “Non-Communicable Diseases – A race against time.”

[17] D. J. Stein *et al.*, “Lifetime prevalence of psychiatric disorders in South Africa.,” *Br. J. Psychiatry*, vol. 192, pp. 112–117, 2008.

[18] B. D. Fulton, R. M. Scheffler, S. P. Sparkes, E. Y. Auh, M. Vujicic, and A. Soucat, “Health




For peer review only - http://bmjopen.bmj.com/site/about/guidelines.xhtml

workforce skill mix and task shifting in low income countries: a review of recent evidence.," *Hum. Resour. Health*, vol. 9, no. 1, p. 1, 2011.

[19] WHO, "Treat, Train, Retain: Task Shifting Global Recomendations and Guidelines," *World Heal. Organ.*, p. 96, 2008.

[20] WHO, "mhGAP Intervention Guide for mental, neurological and substance use disorders in non-specialized health settings Version 2.0," *mhGAP Newsl.*, vol. 1, no. 9, pp. 1–4, 2015.

[21] B. McPake and K. Mensah, "Task shifting in health care in resource-poor countries," *Lancet*, vol. 372, no. 9642, pp. 870–871, 2008.

[22] V. Patel, "Global mental health: From science to action," *Harv. Rev. Psychiatry*, vol. 20, no. 1, pp. 6–12, 2012.

[23] N. van Ginneken *et al.*, "Non-specialist health worker interventions for the care of mental, neurological and substance-abuse disorders in low- and middle-income countries.," *Cochrane database Syst. Rev.*, vol. 11, p. CD009149, 2013.

[24] D. Javadi, I. Feldhaus, A. Mancuso, and A. Ghaffar, "Applying systems thinking to task shifting for mental health using lay providers: a review of the evidence," *Glob. Ment. Heal.*, vol. 4, p. e14, 2017.

[25] T. a. Bruckner *et al.*, "The mental health workforce gap in low- and middle-income countries: A needs-based approach," *Bull. World Health Organ.*, vol. 89, no. 3, pp. 184–194, 2011.

[26] H. Jack *et al.*, "Closing the mental health treatment gap in South Africa: A review of costs and cost-effectiveness," *Glob. Health Action*, vol. 7, no. SUPP.1, pp. 1–11, 2014.

[27] H. Jack, A. Stein, C. R. Newton, and K. J. Hofman, "Expanding access to mental health care: A missing ingredient," *Lancet Glob. Heal.*, vol. 2, no. 4, pp. e183–e184, 2014.

[28] J. Mugisha *et al.*, "Health Systems Context(s) for Integrating Mental Health into Primary Health Care in Six Emerald Countries: A Situation Analysis," *Int. J. Ment. Health Syst.*, vol. 11, no. 1, p. 7, 2017.

[29] J. E. Haberer *et al.*, "Improving antiretroviral therapy adherence in resource-limited settings at scale: A discussion of interventions and recommendations," *J. Int. AIDS Soc.*, vol. 20, no. 1, pp. 1–15, 2017.

[30] F. L. H. Chuah *et al.*, "Interventions and approaches to integrating HIV and mental health services: a systematic review," *Health Policy Plan.*, 2017.

[31] R. Joshi *et al.*, "Task Shifting for Non-Communicable Disease Management in Low and Middle Income Countries – A Systematic Review," *PLoS One*, vol. 9, no. 8, p. e103754, 2014.

[32] B. Myers *et al.*, "Comparing dedicated and designated models of integrating mental health into chronic disease care : study protocol for a cluster randomized controlled trial," pp. 1–13, 2018.

[33] National Department of Health Republic of South Africa, "National Mental Health Policy Framework and Strategic Plan 2013-2020," Pretoria, 2013.

[34] K. Sorsdahl *et al.*, "Adapting a blended motivational interviewing and problem-solving intervention to address risky substance use amongst South Africans.," *Psychother. Res.*, vol. 3307, no. April, pp. 37–41, 2014.

[35] N. T. Van Dam and M. Earleywine, "Validation of the Center for Epidemiologic Studies



For peer review only - http://bmjopen.bmj.com/site/about/guidelines.xhtml

Depression Scale-Revised (CESD-R): Pragmatic depression assessment in the general population," *Psychiatry Res.*, vol. 186, no. 1, pp. 128–132, 2011.

[36] T. F. Babor, J. C. Higgins-Biddle, J. B. Saunders, and M. G. Monteiro, "The Alcohol Use Disorders Identification Test Guidelines for Use in Primary Care," *World Heal. Organ.*, pp. pp1-40, 2001.

[37] R. Rabin and F. De Charro, "EQ-5D: A measure of health status from the EuroQol Group," *Ann. Med.*, vol. 33, no. 5, pp. 337–343, 2001.

[38] W. Hollingworth, D. McKell-Redwood, L. Hampson, and C. Metcalfe, "Cost-utility analysis conducted alongside randomized controlled trials: Are economic end points considered in sample size calculations and does it matter?," *Clin. Trials*, vol. 10, no. 1, pp. 43–53, 2013.

[39] T. Wilkinson *et al.*, "The International Decision Support Initiative Reference Case for Economic Evaluation: An Aid to Thought," *Value Heal.*, vol. 19, no. 8, pp. 921–928, 2016.

[40] D. Walker and L. Kumaranayake, "Allowing for differential timing in cost analyses: discounting and annualization.," *Health Policy Plan.*, vol. 17, no. 1, pp. 112–118, 2002.

[41] M. F. Drummond, M. J. Sculpher, K. Claxton, G. L. Stoddart, and G. W. Torrance, *Methods for the Economic Evaluation of Health Care Programmes*, Fourth. Oxford: Oxford University Press, 2015.

[42] P. Räsänen, E. Roine, H. Sintonen, V. Semberg-Konttinen, O.-P. Ryynanen, and R. Roine, "Use of quality-adjusted life years for the estimation of effectiveness of health care: A systematic literature review," *Int. J. Tech. Assess. Heal. care*, vol. 2, pp. 235–241, 2006.

[43] J. Jelsma, S. Mkoka, L. Amosun, and J. Nieuwveldt, "The reliability and validity of the Xhosa version of the EQ-5D.," *Disabil. Rehabil.*, vol. 26, no. 2, pp. 103–108, 2004.

[44] M. van Reenen and M. Oppe, "EQ-5D-3L User Guide," *EuroQol Res. Found.*, no. April, p. 22, 2015.

[45] S. Kularatna, J. A. Whitty, N. W. Johnson, and P. A. Scuffham, "Health State Valuation in Low- and Middle-Income Countries: A Systematic Review of the Literature," *Value Heal.*, vol. 16, no. 6, pp. 1091–1099, 2013.

[46] M. F. Drummond, M. J. Sculpher, K. Claxton, G. L. Stoddart, and G. W. Torrance, *Methods for the Economic Evaluation of Health Care Programmmes*, 4th ed. Oxford: Oxford University Press, 2015.

[47] C. Brooke-Sumner, K. Sorsdahl, C. Lombard, P. Petersen-Williams, and B. Myers, "Protocol for development and validation of a context-appropriate tool for assessing organisational readiness for change in primary health clinics in South Africa," *BMJ Open*, vol. 8, no. 4, p. e020539, 2018.

[48] J. A. Hontelez, M.-L. Newell, R. M. Bland, K. Munnelly, R. J. Lessells, and T. Bärnighausen, "Human resources needs for universal access to antiretroviral therapy in South Africa: a time and motion study," *Hum. Resour. Health*, vol. 10, no. 1, p. 39, 2012.

[49] H. Naburi *et al.*, "The potential of task-shifting in scaling up services for prevention of mother-to-child transmission of HIV: a time and motion study in Dar es Salaam, Tanzania.," *Hum. Resour. Health*, vol. 15, no. 1, p. 35, 2017.

[50] W. A. Odendaal and S. Lewin, "The provision of TB and HIV/AIDS treatment support by lay health workers in South Africa: a time-and-motion study.," *Hum. Resour. Health*, vol. 12, no. 1, p. 18, 2014.



For peer review only - http://bmjopen.bmj.com/site/about/guidelines.xhtml

[51] L. Cunnama *et al.*, "Using Top-down and Bottom-up Costing Approaches in LMICs: The Case for Using Both to Assess the Incremental Costs of New Technologies at Scale," *Health Econ.*, vol. 25, no. January, 2016.

[52] N. Foster, A. Vassall, S. Cleary, L. Cunnama, G. Churchyard, and E. Sinanovic, "The economic burden of TB diagnosis and treatment in South Africa," *Soc. Sci. Med.*, vol. 130, pp. 42–50, 2015.

[53] V. Mauch, N. Woods, B. Kirubi, H. Kipruto, J. Sitienei, and E. Klinkenberg, "Assessing access barriers to tuberculosis care with the tool to Estimate Patients' Costs: Pilot results from two districts in Kenya," *BMC Public Health*, vol. 11, pp. 18–21, 2011.

[54] L. Ramma *et al.*, "Patients' costs associated with seeking and accessing treatment for drug-resistant tuberculosis in South Africa," *Int. J. Tuberc. Lung Dis.*, vol. 19, no. 12, pp. 1513–1519, 2015.

[55] D. Husereau *et al.*, "Consolidated Health Economic Evaluation Reporting Standards (CHEERS) statement," *Eur. J. Heal. Econ.*, vol. 14, no. 3, pp. 367–372, 2013.

[56] J. Karnon and J. Brown, "Selecting a decision model for economic evaluation: A case study and review," *Health Care Manag. Sci.*, vol. 1, pp. 133–140, 1998.

[57] P. Thokala, J. Ochalek, A. A. Leech, and T. Tong, "Cost-Effectiveness Thresholds: the Past, the Present and the Future," *Pharmacoeconomics*, pp. 1–14, 2018.

[58] A. M. Gray and T. Wilkinson, "Economic evaluation of healthcare interventions: Old and new directions," *Oxford Rev. Econ. Policy*, vol. 32, no. 1, pp. 102–121, 2016.

[59] A. Manca, N. Hawkins, and M. J. Sculpher, "Estimating mean QALYs in trial-based cost-effectiveness analysis: The importance of controlling for baseline utility," *Health Econ.*, vol. 14, no. 5, pp. 487–496, 2005.

[60] J. L. Severens, R. J. Milne, and H. Severens, "Discounting health outcomes in economic evaluation: The ongoing debate," *Value Heal.*, vol. 7, no. 4, pp. 397–401, 2004.

[61] A. E. Attema, W. B. F. Brouwer, and K. Claxton, "Discounting in Economic Evaluations," *Pharmacoeconomics*, vol. 36, no. 7, pp. 745–758, 2018.

[62] R. Dwommoh *et al.*, "Brief interventions to address substance use among patients presenting to emergency departments in resource poor settings : a cost ‐ effectiveness analysis," *Cost Eff. Resour. Alloc.*, pp. 1–11, 2018.

[63] D. Hughes *et al.*, "Conducting Economic Evaluations Alongside Randomised Trials: Current Methodological Issues and Novel Approaches," *Pharmacoeconomics*, vol. 34, no. 5, pp. 447–461, 2016.

[64] D. A. Richards *et al.*, "Clinical effectiveness of collaborative care for depression in UK primary care (CADET): cluster randomised controlled trial," *BMJ*, vol. 347, no. 14, p. f4913, 2013.

[65] W. Katon *et al.*, "Cost-effectiveness of a multicondition collaborative care intervention," *Arch. Gen. Psychiatry*, vol. 69, no. 5, pp. 506–514, 2012.

[66] J. W. Hay, W. J. Katon, K. Ell, P.-J. Lee, and J. J. Guterman, "Cost-Effectiveness Analysis of Collaborative Care Management of Major Depression among Low-Income, Predominantly Hispanics with Diabetes," *Value Heal.*, vol. 15, no. 2, pp. 249–254, 2012.

[67] M. Funk and G. Ivbijaro, "Integrating mental health and primary care: A global perspective," *World Health Organisation*, 2008. .



For peer review only - http://bmjopen.bmj.com/site/about/guidelines.xhtml

[68] S. Baine and A. Kasangaki, "A scoping study on task shifting; the case of Uganda," *BMC Health Serv. Res.*, vol. 14, no. 1, p. 184, 2014.

[69] V. Patel, D. Chisholm, S. Rabe-Hesketh, F. Dias-Saxena, G. Andrew, and A. Mann, "Efficacy and cost-effectiveness of drug and psychological treatments for common mental disorders in general health care in Goa, India: a randomised, controlled trial.," *Lancet (London, England)*, vol. 361, no. 9351, pp. 33–9, 2003.

[70] D. Siskind, F. K. Baingana, and J. Kim, "Cost-Effectiveness of Group Psycotherapy Depression For Depression in Uganda," *J. Ment. Heal. Policy Econ.*, vol. 11, pp. 127–133, 2008.

[71] S. Cleary, G. Mooney, and D. Mcintyre, "EQUITY AND EFFICIENCY IN HIV-TREATMENT IN SOUTH AFRICA: THE CONTRIBUTION OF MATHEMATICAL PROGRAMMING TO PRIORITY SETTING SUSAN," *Health Econ.*, vol. 19, pp. 1166–1180, 2010.

[72] A. Bhana, I. Petersen, K. L. Baillie, A. J. Flisher, and The Mhapp Research Programme Consortium, "Implementing the World Health Report 2001 recommendations for integrating mental health into primary health care: a situation analysis of three African countries: Ghana, South Africa and Uganda.," *Int. Rev. Psychiatry*, vol. 22, pp. 599–610, 2010.

[73] P. F. Santos, M. L. Wainberg, J. M. Caldas-de-Almeida, B. Saraceno, and J. de J. Mari, "Overview of the mental health system in Mozambique: Addressing the treatment gap with a task-shifting strategy in primary care," *Int. J. Ment. Health Syst.*, vol. 10, no. 1, pp. 1–9, 2016.

[74] S. Jan, "A holistic approach to the economic evaluation of health programs using institutionalist methodology.," *Soc. Sci. Med.*, vol. 47, no. 10, pp. 1565–1572, 1998.

[75] S. Jan, "Institutional considerations in priority setting: transactions cost perspective on PBMA.," *Health Econ.*, vol. 9, no. 7, pp. 631–41, 2000.

[76] S. Jan, P. Pronyk, and J. Kim, "Accounting for institutional change in health economic evaluation: a program to tackle HIV/AIDS and gender violence in Southern Africa.," *Soc. Sci. Med.*, vol. 66, no. 4, pp. 922–32, 2008.

[77] M. Spedding, D. Stein, and K. S. A. H. R. 2014; 15: 73-87. Sorsdahl, "Task-shifting psychosocial interventions in public mental health: A review of the evidence in the South African context.," *South African Heal. Rev. 2014/2015*, vol. 15, pp. 73–84, 2015.

[78] I. Petersen, C. Lund, A. Bhana, and A. J. Flisher, "A task shifting approach to primary mental health care for adults in South Africa: Human resource requirements and costs for rural settings," *Health Policy Plan.*, vol. 27, no. 1, pp. 42–51, 2012.

[79] R. Atun, T. De Jongh, F. Secci, K. Ohiri, and O. Adeyi, "Integration of targeted health interventions into health systems: A conceptual framework for analysis," *Health Policy Plan.*, vol. 25, no. 2, pp. 104–111, 2010.

[80] A. Shigayeva, R. Atun, M. McKee, and R. Coker, "Health systems, communicable diseases and integration," *Health Policy Plan.*, vol. 25, no. Suppl. 1, pp. i4–i20, 2010.

[81] L. Dudley and P. Garner, "Strategies for integrating primary health services in low- and middle-income countries at the point of delivery.," *Cochrane Database Syst. Rev.*, no. 2, p. CD003318, 2011.

[82] C. Van Deventer, "Integration of non-communicable chronic diseases ( NCDs ) and HIV / AIDS and mental health care through the involvement of chronically ill patients using empowerment evaluation Integration of non-communicable chronic diseases ( NCDs ) and HIV / AIDS and m," vol. 6190, no. January 2016, pp. 337–346, 2015.



For peer review only - http://bmjopen.bmj.com/site/about/guidelines.xhtml

[83] V. Maizes, D. Rakel, and C. Niemiec, "Integrative Medicine and Patient-Centered Care," *Explor. J. Sci. Heal.*, vol. 5, no. 5, pp. 277–289, 2009.

[84] Western Cape Government, "Healthcare 2030: The Road to Wellness," Cape Town, 2014.

[85] A. J. Cowell, J. M. Brown, M. J. Mills, R. H. Bender, and B. J. Wedehase, "Cost-effectiveness analysis of motivational interviewing with feedback to reduce drinking among a sample of college students," *J. Stud. Alcohol Drugs*, vol. 73, no. 2, pp. 226–37, 2012.

[86] A. J. Cowell, J. W. Bray, M. J. Mills, and J. M. Hinde, "Conducting economic evaluations of screening and brief intervention for hazardous drinking: Methods and evidence to date for informing policy," *Drug Alcohol Rev.*, vol. 29, no. 6, pp. 623–630, 2010.

[87] M. C. Weinstein, G. Torrance, and A. Mcguire, "QALYs : The Basics," vol. 12, 2009.

[88] T. Powell-Jackson *et al.*, "Trials and tribulations: cross-learning from the practices of epidemiologists and economists in the evaluation of public health interventions," *Health Policy Plan.*, no. May, pp. 1–5, 2018.

[89] C. Lund *et al.*, "Poverty and common mental disorders in low and middle income countries: A systematic review.," *Soc. Sci. Med.*, vol. 71, no. 3, pp. 517–528, 2010.

[90] H. A. Glick, J. A. Doshi, S. S. Sonnad, and D. Polsky, *Economic Evauation in Clinical Trials*, 2nd ed. New York: Oxford University Press, 2015.

[91] L. Rispel, J. Moorman, M. Chersich, J. Goudge, N. Nxumalo, and T. Ndou, "REVITALISING PRIMARY HEALTH CARE IN SOUTH AFRICA: Review of primary health care package, norms and standards," Johannesburg, 2010.

For peer review only



For peer review only - http://bmjopen.bmj.com/site/about/guidelines.xhtml

| Analysis | *Assessment/ Economic Evaluation Outcomes* | Measurement instrument OR Clinical Parameter | Assessment Timing | | | |
|---|---|---|---|---|---|---|
| | | Intervention start → | Baseline | 6 months | 1 Year | ← Intervention assessment end |
| | ***1º Outcomes*** | | | | | |
| CEA | Harmful alcohol use (Self-report interview) | AUDIT | ✓ | ✓ | ✓ | |
| CEA | Depression (Self-report interview) | CES-D | ✓ | ✓ | ✓ | |
| CEA | ***2º Outcomes*** | | | | | |
| CEA | Diabetes treatment failure risk | HbA1c | ✓ | ✓ | ✓ | |
| CEA | | BMI | | | | |
| CEA | HIV treatment failure risk | HIV-1 RNA (HIV-viral load) | ✓ | ✓ | ✓ | |
| CEA | Chronic Disease Medication Adherence | Visual analogue Scale and CASE adherence Index | ✓ | ✓ | ✓ | |
| CUA | ***Multi-attribute Outcomes*** | | | | | |
| CUA | QALY | EQ-5D | ✓ | ✓ | ✓ | |

**Figure 1** Economic Evaluation: Analyses, Outcomes, Measurement and Assessment Timing

For peer review only

**CHEERS checklist**
**Items to include when reporting economic evaluations of health interventions**

Further discussion of the checklist may be accessed from the ISPOR Health Economic Evaluation Publication Guidelines – CHEERS: Good Reporting Practices webpage: http://www.ispor.org/TaskForces/EconomicPubGuidelines.asp.

| Section /item | Item No | Recommendation | Reported on page No/ line No |
|---|---|---|---|
| **Title and abstract** | | | |
| Title | 1 | Identify the study as an economic evaluation or use more specific terms such as "cost-effectiveness analysis", and describe the interventions compared. | Page 1 |
| Abstract | 2 | Provide a structured summary of objectives, perspective, setting, methods (including study design and inputs), results (including base case and uncertainty analyses), and conclusions. | Page 2 |
| **Introduction** | | | |
| Background and objectives | 3 | Provide an explicit statement of the broader context for the study. | Page 3 |
| | | Present the study question and its relevance for health policy or practice decisions. | Page 5 |
| **Methods** | | | |
| Target population and subgroups | 4 | Describe characteristics of the base case population and subgroups analysed, including why they were chosen. | Page 5 |
| Setting and location | 5 | State relevant aspects of the system(s) in which the decision(s) need(s) to be made. | Page 4 |
| Study perspective | 6 | Describe the perspective of the study and relate this to the costs being evaluated. | Page 6 |
| Comparators | 7 | Describe the interventions or strategies being compared and state why they were chosen. | Page 5 |
| Time horizon | 8 | State the time horizon(s) over which costs and consequences are being evaluated and say why appropriate. | Page 6 |
| Discount rate | 9 | Report the choice of discount rate(s) used for costs and outcomes and say why appropriate. | Page 6 |
| Choice of health outcomes | 10 | Describe what outcomes were used as the measure(s) of benefit in the evaluation and their relevance for the type of analysis performed. | Page 6 |
| Measurement of effectiveness | 11a | *Single study-based estimates:* Describe fully the design features of the single effectiveness study and why the single study was a sufficient source of clinical effectiveness data. | Page 6 |
| | 11b | *Synthesis-based estimates*: Describe fully the methods used for identification of included studies and synthesis of clinical effectiveness data. | |
| Measurement and valuation of preference based outcomes | 12 | If applicable, describe the population and methods used to elicit preferences for outcomes. | Page 6 |
| Estimating resources and costs | 13a | *Single study-based economic evaluation:* Describe approaches used to estimate resource | Page 6 |

For peer review only - http://bmjopen.bmj.com/site/about/guidelines.xhtml

| Section /item | Item No | Recommendation | Reported on page No/ line No |
|---|---|---|---|
| | | use associated with the alternative interventions. Describe primary or secondary research methods for valuing each resource item in terms of its unit cost. Describe any adjustments made to approximate to opportunity costs. | |
| | 13b | *Model-based economic evaluation:* Describe approaches and data sources used to estimate resource use associated with model health states. Describe primary or secondary research methods for valuing each resource item in terms of its unit cost. Describe any adjustments made to approximate to opportunity costs. | Not applicable |
| Currency, price date, and conversion | 14 | Report the dates of the estimated resource quantities and unit costs. Describe methods for adjusting estimated unit costs to the year of reported costs if necessary. Describe methods for converting costs into a common currency base and the exchange rate. | Page 8 |
| Choice of model | 15 | Describe and give reasons for the specific type of decision-analytical model used. Providing a figure to show model structure is strongly recommended. | Protocol – not applicable |
| Assumptions | 16 | Describe all structural or other assumptions underpinning the decision-analytical model. | Protocol – not applicable |
| Analytical methods | 17 | Describe all analytical methods supporting the evaluation. This could include methods for dealing with skewed, missing, or censored data; extrapolation methods; methods for pooling data; approaches to validate or make adjustments (such as half cycle corrections) to a model; and methods for handling population heterogeneity and uncertainty. | Page 10 |
| **Results** | | | |
| Study parameters | 18 | Report the values, ranges, references, and, if used, probability distributions for all parameters. Report reasons or sources for distributions used to represent uncertainty where appropriate. Providing a table to show the input values is strongly recommended. | Protocol – not applicable |
| Incremental costs and outcomes | 19 | For each intervention, report mean values for the main categories of estimated costs and outcomes of interest, as well as mean differences between the comparator groups. If applicable, report incremental cost-effectiveness ratios. | Protocol – not applicable |
| Characterising uncertainty | 20a | *Single study-based economic evaluation:* Describe the effects of sampling uncertainty for the estimated incremental cost and incremental effectiveness parameters, together with the impact of methodological assumptions (such as discount rate, study perspective). | Protocol – not applicable |
| | 20b | *Model-based economic evaluation:* Describe the effects on the results of uncertainty for all input parameters, and uncertainty related to the | Not applicable |

For peer review only

| Section /item | Item No | Recommendation | Reported on page No/ line No |
|---|---|---|---|
| | | structure of the model and assumptions. | |
| Characterising heterogeneity | 21 | If applicable, report differences in costs, outcomes, or cost-effectiveness that can be explained by variations between subgroups of patients with different baseline characteristics or other observed variability in effects that are not reducible by more information. | Protocol – not applicable |
| **Discussion** | | | |
| Study findings, limitations, generalisability, and current knowledge | 22 | Summarise key study findings and describe how they support the conclusions reached. Discuss limitations and the generalisability of the findings and how the findings fit with current knowledge. | Protocol discussion |
| **Other** | | | |
| Source of funding | 23 | Describe how the study was funded and the role of the funder in the identification, design, conduct, and reporting of the analysis. Describe other non-monetary sources of support. | Page 12 |
| Conflicts of interest | 24 | Describe any potential for conflict of interest of study contributors in accordance with journal policy. In the absence of a journal policy, we recommend authors comply with International Committee of Medical Journal Editors recommendations. | Information provided via the submission system |

For consistency, the CHEERS statement checklist format is based on the format of the CONSORT statement checklist

The citation for the CHEERS Task Force Report is: Husereau D, Drummond M, Petrou S, et al. Consolidated health economic evaluation reporting standards (CHEERS)—Explanation and elaboration: A report of the ISPOR health economic evaluations publication guidelines good reporting practices task force. Value Health 2013;16:231-50.

For peer review only

BMJ Open

# Integrating a Brief Mental Health Intervention into Primary Care Services for HIV and Diabetes Patients in South Africa: Study Protocol for a Trial Based Economic Evaluation.

| | |
|---|---|
| Journal: | *BMJ Open* |
| Manuscript ID | bmjopen-2018-026973.R1 |
| Article Type: | Protocol |
| Date Submitted by the Author: | 26-Dec-2018 |
| Complete List of Authors: | Mutyambizi-Mafunda, Vimbayi; University of Cape Town School of Public Health and Family Medicine, Health Economics Unit;<br>Myers, Bronwyn; South African Medical Research Council, Alcohol and Drug Abuse Research Unit<br>Sorsdahl, Katherine<br>Lund, Crick; University of Cape Town, Alan J Flisher Centre for Public Mental Health<br>Naledi, Tracey; University of Cape Town<br>Cleary, S; University of Cape Town, Health Economics Unit |
| <b>Primary Subject Heading</b>: | Health economics |
| Secondary Subject Heading: | Mental health |
| Keywords: | cost-effectiveness analysis, common mental disorders, HIV, Diabetes, integrated treatment |
| | |

SCHOLARONE™
Manuscripts

# Integrating a Brief Mental Health Intervention into Primary Care Services for HIV and Diabetes Patients in South Africa: Study Protocol for a Trial Based Economic Evaluation.

**Authors:** Vimbayi Mutyambizi-Mafunda[1], Bronwyn Myers[2,3], Katherine Sorsdahl[4], Crick Lund[4,5], Tracey Naledi[6], Susan Cleary[1]

[1] Health Economics Unit, School of Public Health and Family Medicine, University of Cape Town, Cape Town, South Africa.

[2] Alcohol, Tobacco and Other Drug Research Unit, South African Medical Research Council, Francie van Zyl Drive, Tygerberg, Cape Town, 7505, South Africa

[3] Division of Addiction Psychiatry, Psychiatry and Mental Health, University of Cape Town, Cape Town, South Africa.

[4]Alan J Flisher Centre for Public Mental Health, Department of Psychiatry and Mental Health, University of Cape Town, Cape Town, South Africa.

[5] Centre for Global Mental Health, Institute of Psychiatry, Psychology and Neuroscience, King's College, London, UK.

[6] Western Cape Department of Health, 8 Riebeeck Street, Cape Town, South Africa.

**Author Email addresses:**

Vimbayi Mutyambizi-Mafunda - vimbayi.mafunda@uct.ac.za

Bronwyn Myers – Bronwyn.Myers@mrc.ac.za

Katherine Sorsdahl – katherine.sorsdahl@uct.ac.za

Crick Lund – crick.lund@uct.ac.za

Tracey Naledi – Tracey.Naledi@hiv-research.org.za

Susan Cleary – susan.cleary@uct.ac.za

**Corresponding Author**

**Name:** Vimbayi Mutyambizi-Mafunda

**Postal Address:** Health Economics Unit, University of Cape Town, School of Public Health & Family Medicine, Health Sciences Faculty, Anzio Road, Observatory, Cape Town,7925, South Africa.

**Email address:** vimbayi.mafunda@uct.ac.za

**Telephone Number:** +27 21 406 6558

**Word count:** excluding abstract and article summary - 4714

For peer review only



For peer review only - http://bmjopen.bmj.com/site/about/guidelines.xhtml

## ABSTRACT

**Introduction:** Depression and alcohol use disorders are international public health priorities for which there is a substantial treatment gap. Brief mental health interventions delivered by lay health workers in primary care services may reduce this gap. There is limited economic evidence assessing the cost-effectiveness of such interventions in low-and middle-income countries (LMIC). This paper describes the proposed economic evaluation of a health systems intervention testing the effectiveness and cost-effectiveness of two task-sharing approaches to integrating services for common mental disorders with HIV and Diabetes primary care services.

**Methods and analysis:** This evaluation will be conducted as part of a three-armed cluster-randomised controlled trial of clinical effectiveness. Trial clinical outcome measures will include primary outcomes for risk of depression and alcohol use, and secondary outcomes for risk of chronic disease (HIV and Diabetes) treatment failure. The cost effectiveness analysis will evaluate cost per unit change in AUDIT and CES-D scores as well as cost per unit change in HIV RNA viral load and HBA1c, producing results of provider and patient cost per patient year for each study arm and chronic disease. The cost utility analyses will provide results of cost per quality adjusted life year (QALY) gained. Additional analyses relevant for implementation including budget impact analyses will be conducted to inform the development of a business case for scaling up the country's investment in mental health services.

**Ethics and dissemination:** The Western Cape Department of Health (WCDoH) (WC2016_RP6_9), the South African Medical Research Council (EC 004-2/2015), the University of Cape Town (089/2015), and Oxford University (OxTREC 2-17) provided ethical approval for this study. Results dissemination will be through policy briefs, social media, peer-reviewed papers, a policy dialogue workshop to engage with policy makers and press briefings for national and local media.

**Trial registration**: Pan African Clinical Trials registry (registration number PACTR201610001825405).


**Keywords:** cost-effectiveness analysis, common mental disorders, HIV, Diabetes, integrated treatment

## ARTICLE SUMMARY

### Strengths and limitations of this study

- This study will provide some of the first empirical evidence of the cost-effectiveness of integrating services for common mental disorders in the primary care offering for HIV and Diabetes.
- The study will generate findings that may guide priority setting through application of QALY based outcomes and decision-making around the implementation of mental health counselling within the health system.
- This paper adds to the growing body of protocols for economic evaluations conducted alongside RCTs.
- As health service use is self-reported, there is a possibility for recall bias, to limit this, a one month recall period will be used for ambulatory services and a 6 month recall for hospitalisation, and hospitalisations will be validated through the Department of Health Data Centre.
- The one year follow up period may reflect outcomes that do not fully represent the full benefit that patients and the health system could gain through the availability of this service; further work using an extended follow-up period may be proposed.



For peer review only - http://bmjopen.bmj.com/site/about/guidelines.xhtml

## INTRODUCTION

Common mental disorders (CMDs) including depression, anxiety and alcohol use disorders are highly prevalent conditions that impose a significant societal cost and impact on quality of life [1]. Globally, these conditions have been shown to be the leading cause of non-fatal burden of disease [1], [2]. The disabling impact of these conditions is particularly significant in patients suffering from chronic physical conditions [3]. CMD co-morbidity has been shown to: exacerbate major modifiable risk factors for chronic disease; contribute to chronic disease progression; increase the prevalence of preventable complications and disability; result in poor adherence; and ultimately lead to treatment failure [1], [4]. There is a plethora of evidence on the impact of depression and alcohol use disorders (AUD) on diabetes outcomes [5], [6]. Likewise, alcohol consumption and depressive conditions have been associated with poor HIV outcomes [7], [8]. In addition high-income country (HIC) evidence suggests that patients with these multi-morbidities have higher health care costs even after adjusting for the costs of mental health care [9]; suggesting potential cost savings from investing in mental health treatment for patients with multi-morbidities[10].

There is a pressing need for evidence on cost-effective strategies for integrating mental health care into primary health services, particularly in low-and middle-income countries (LMICs) where health systems are typically fragile and chronically underfunded. In these health systems, an investment in mental health services at primary care level for chronic disease patients has the potential to result in significant returns on investment due to reduced chronic disease treatment costs. South Africa - with the world's largest ART programme [11], an increasing prevalence of diabetes [11] and a huge unmet need for CMD treatment [12] - is one LMIC that may benefit from this integrated approach.

Task-sharing [13], [14] involves re-distributing tasks from specialist providers to non-specialist health workers [15] with specialists providing on-going support to these health workers. Task-sharing is a common strategy for the provision of primary care services in constrained LMIC settings [16]. Due to the long lead times in training mental health staff and financial resource limitations, the task-sharing discourse has found appeal in the policy discussions around introducing and expanding mental health care in LMICs [1], [17]. These discussions are centred on using task-shared approaches [18] to upskill lay health workers to integrate mental health services such as brief counselling interventions into primary care [19], [20].Task-sharing the psychosocial counselling of patients who have chronic co-morbidities to lay health workers is a particularly appealing and potentially cost-effective strategy to treat patients and support their adherence to chronic medication [21], [22] and therefore limit overall health system costs of care [4], [16].

While task-sharing the treatment for CMDs from mental health professionals to lower level health care workers has been suggested as an effective way to deliver services within the context of scarce human resources [4] and has been shown to be cost effective in some HIC settings, there is little if any economic evidence to support the implementation of such strategies in LMICs [4], [16]. This lack of evidence limits decision making around the allocation of resources for these services. Economic evidence on the institutional investment, cost-effectiveness and budget impact of this approach is necessary for empirically-informed health service planning [23]. This work presents a protocol for a cost-effectiveness and cost-utility analysis from a societal (i.e. provider and patient perspective) of a task-sharing strategy for delivering a brief mental health counselling intervention to patients with comorbid HIV or Diabetes in South Africa.



For peer review only - http://bmjopen.bmj.com/site/about/guidelines.xhtml

## METHODS AND ANALYSIS

### Trial design

This study is nested in Project MIND, a cluster randomised control trial across 24 public primary care facilities [24]. Myers et al., (2018) describes the trial in detail [24].The trial will compare the effectiveness of treatment as usual (MIND_TAU) and two alternative methods for integrating a psychosocial intervention into the primary care services for HIV and Diabetes. Treatment as usual (TAU) is representative of typical primary care level services for CMD in the South African public health service facilities, which is typically limited to referrals [25],[26]. The two models will be the dedicated and designated models of care. In the dedicated approach (MIND_DED), community health workers (CHWs) will be hired and added to the facility staff complement and will dedicate their time to *only* delivering the new counselling service. In the designated approach (MIND_DES) CHWs already working in the facility will be designated to deliver the service *in addition* to their other chronic disease-related activities such as adherence counselling for HIV and health promotion. Supervision and de-briefing of all the counsellors in the active arms will be task-shifted to registered psychological counsellors in line with the national mental health policy framework [27]. Descriptions of the CHWs and registered psychological counsellors roles in the MIND-DED and MIND-DES models and their qualifications and skills levels are detailed in the trial protocol [24]. The Western Cape Department of Health (WCDoH) (WC2016_RP6_9), the South African Medical Research Council (EC 004-2/2015), the University of Cape Town (089/2015), and Oxford University (OxTREC 2-17) approved the study.

### *Intervention and comparator*

The counselling programme delivered in MIND-DED and MIND-DES is based on Motivational Interviewing (MI) and Problem Solving Therapy (PST) [28]. It is delivered in three sessions over six weeks with one optional booster session. The MI component provides psychoeducation about depression and hazardous alcohol use in relation to chronic disease, builds readiness for counselling and helps participants develop goals for counselling. The PST component targets maladaptive coping strategies and builds skills and strategies for dealing with life problems. Patients will receive individual face-to-face counselling from CHWs and take-home booklets to support self-learning between sessions. Registered psychological counsellors, supervised by psychologists, will train the counsellors (see [24] for more detail). The underlying theory, content and evidence for the efficacy of the counselling approach for reducing symptoms of depression and hazardous /harmful alcohol use over the 1 year time frame have been described previously [29]–[31]. In them TAU arm, the standard package of care will be provided to patients who are suspected of having mental health problems. In general, patients using the HIV or diabetes service are asked by a nurse or doctor attending to their care about their mental well-being, life stressors, and use of alcohol or other substances. Patients are provided with advice to change their lifestyles. Where the health care provider deems it necessary, patients are referred to a mental health nurse for further assessment or screening. The patient may also be referred to a social worker who may refer them to NGOs who provide counselling and support services [24].

### *Outcome Measures*

The primary outcomes for the trial are measures of psychosocial functioning, specifically changes in self-reported risk of depression as measured by CES-D (Centre for Epidemiological Studies scale on Depression)[32] and self-reported hazardous alcohol use as measured by the AUDIT (Alcohol Use Disorder Identification Test) [33]. Secondary outcomes include biological measures aimed at assessing changes in chronic disease treatment response, specifically HbA1c for diabetes and HIV-1 RNA viral loads for HIV. Other secondary outcome measures include self-reported adherence to chronic disease medication and health-related quality of life (HRQoL) measured using the EQ-5D [34]. All outcomes



For peer review only - http://bmjopen.bmj.com/site/about/guidelines.xhtml

are measured longitudinally at baseline, and at 6 and 12 month follow-up assessments, recruitment on the trial started in 2017 and will end in February 2019, and final outcome assessments will end in 2020 (see **Figure 1)**.

***Sample Size and Patient Population***

Recruitment will be restricted to consenting adult patients who meet criteria for hazardous alcohol use or depression using the AUDIT or CES-D. These patients are already diagnosed and receiving treatment for diabetes or HIV, but not currently receiving treatment for a CMD. The trial requires a sample size of 1200 patients (600 with HIV and 600 with diabetes) to detect reductions in hazardous alcohol use and risk of depression at 12 month follow-up. The sample size calculations are based on a cluster randomised design with two active arms and a control arm. This clinical trial is powered to detect clinical outcomes, specifically reductions hazardous/ harmful alcohol use and risk of depression at 12-month follow-up rather than economic outcomes [24],[35].

**MIND Economic Evaluation**

**Objective**

The objective of this prospective economic evaluation is to estimate for alternative intervention options the:

i. Full economic provider costs: of the mental health intervention; any cost offsets attributable to the intervention related to changes to the costs of HIV or Diabetes care at the primary care level; and changes to the costs of referral care (including tuberculosis, emergency department and inpatient care);
ii. Patient costs: associated with the intervention which will include direct OOP payments to private health providers (consultations and medications etc.), travel and subsistence costs and productivity losses.
iii. Cost-effectiveness in terms of the incremental cost per unit of improvement in disease specific outcome (CES-D, AUDIT, HIV-1 RNA, HBA1c);
iv. Cost-utility in terms of the incremental cost per Quality Adjusted Life Year (QALY) gained and;
v. Budget impact of scaling up the intervention.

To achieve these objectives, the incremental cost-effectiveness ratio (ICER) will be calculated by ordering the MIND_DED, the MIND_DES and MIND_TAU alternatives from least cost to highest cost and calculating the difference in costs and outcomes against the next less costly, non-dominated alternative or against the common baseline of MIND_TAU.

**Perspective**

The aim of the economic evaluation is to inform health service decision makers about the costs, outcomes, economic efficiencies and budget impact of introducing task-shared psychosocial counselling to their chronic disease patient body in a resource-scarce context. As such, the economic evaluation will be conducted from a provider perspective, reflecting health service budget constraints. In line with good practise recommendations, the analyses will also be presented from a societal perspective (including both provider and patient perspectives). While public sector primary health care is free at the point of use, patients incur time and travel costs when accessing care and may experience productivity losses; in addition, they may incur costs when using private sector health services. These costs will be collected to inform the patient perspective within the economic evaluation.

**Time Horizon and Discount Rate**

As the health consequences of the intervention will be experienced over a period extending longer than one year, a discount rate will be applied to reflect differentials in timing[36]. A three percent



For peer review only - http://bmjopen.bmj.com/site/about/guidelines.xhtml

discount rate will be used for discounting both costs and outcomes to allow comparability with other cost-effectiveness analyses [37].

**Estimating intervention effects**

Single study-based estimates of clinical effectiveness will be obtained from the trial, which will provide the primary and secondary outcome measures previously described. In addition to the disease-specific primary outcomes, and the secondary HIV and diabetes outcomes which will be applied in the cost-effectiveness analysis, the QALY will also be calculated as an end-point that will be used in the cost-utility analysis. The ICER resulting when using a QALY measure represents the additional cost associated with accruing another QALY as one compares strategies (that is, the incremental cost per QALY gained). The QALY allows comparison of ICERs across different diseases and interventions [37]. QALYs are calculated by multiplying the length of time in a health state by the HRQoL of the health state. The EQ-5D is the most widely used instrument for measuring HRQoL for cost-utility analyses [34] . In South Africa it has been validated and translated into Afrikaans and isiXhosa, two of the country's official languages [38]. The EQ-5D is a self-assessment of health status across five domains which include mobility, self-care, usual activities, pain and discomfort and anxiety/depression[39]. In this study, the EQ-5D-3L [34] will be used to measure HRQoL. A single summary index score will be generated using preference weights derived from a time trade-off (TTO) based valuation survey conducted in Zimbabwe in addition to the commonly used United Kingdom value set; the TTO is the most common valuation technique used in LMICs [40]. As the EQ-5D will be administered at all the assessment intervals (baseline, 6 months and 12-months post-enrolment) there will be three observations of the EQ-5D scores for each patient in the trial. The QALY gained per patient over the trial period will be calculated by measuring the area under the quality of life curve[37].

**Estimating costs for the provider's perspective**

Within economic evaluation, the appropriate scope of provider costs includes all costs incurred within the intervention and any changes in broader health system costs that can be attributable to the intervention. We have categorized these costs as intervention costs, HIV and Diabetes service costs and other related provider costs. As shown in **Table 1**, our approach to estimating these costs entails the measurement of quantities of resources that are utilized, and multiplying these quantities by the value (or unit cost) of each resource. These separate steps of measurement and valuation are described below.

| | **MEASUREMENT** | | **VALUATION** | |
|---|---|---|---|---|
| **Cost component** | **Resources used** | **Quantities or utilisation** | **Valuation data** | **Allocation factor** |
| ***INTERVENTION*** | | | | |
| *Capital costs* | | | | |
| Facility Readiness Workshops | Number of staff | Time from trial data | Cost of Employment converted to annual equivalent cost | DED/DES/TAU headcount |
| Intervention training | Number of staff | Time from trial data | Cost of Employment converted to annual equivalent cost | DED/DES/TAU headcount |
| Counselling room | Space | Square meters | Replacement value converted to annual equivalent cost | DED/DES/TAU headcount |
| Furniture & equipment | e.g. Tape recorders | Inventory | Replacement value converted to annual equivalent cost | DED/DES/TAU headcount |



For peer review only - http://bmjopen.bmj.com/site/about/guidelines.xhtml

| | | | | |
|---|---|---|---|---|
| Vehicles | Vehicles | Inventory | Replacement value converted to annual equivalent cost | DED/DES/TAU headcount |
| *Recurrent costs* | | | | |
| Counselling personnel | Number of staff (Lay counsellors; registered counsellors; clinical psychologist) | Time from time and motion tool | Cost of Employment X proportion of time | DED/DES/TAU headcount |
| Counselling supplies | Manuals, notebooks, pens | Number used - from trial data | Market value | DED/DES/TAU headcount |
| Non-intervention personnel | Number of staff | Time from facility data | Cost of Employment X proportion of time | Facility headcount |
| Utilities | Electricity, water, other utilities, phone, cleaning, transport, and stationery | Facility utilisation | Annual facility expenditure | Facility headcount |
| ***HIV/DIABETES SERVICE*** | | | | |
| *Capital costs* | | | | |
| Buildings | Space used for HIV/Diabetes service | Square meters | Replacement value converted to annual equivalent cost | HIV/Diabetes headcount |
| Equipment& furniture | Equipment and furniture used for HIV/Diabetes service | Inventory | Replacement value converted to annual equivalent cost | HIV/Diabetes headcount |
| Vehicles | Vehicles used for HIV/Diabetes service | Inventory | Replacement value converted to annual equivalent cost | HIV/Diabetes headcount |
| *Recurrent costs* | | | | |
| HIV/Diabetes personnel | Number of staff and staff time spent on HIV/Diabetes service | Time from facility data | Cost of Employment X proportion of time | HIV/Diabetes headcount |
| Drugs | ARV/Diabetes medication | Treatment protocol | National tender prices | Direct allocation |
| Diagnostic tests | CD4 test, diabetes monitoring tests etc. | Treatment protocol | National Health Laboratory Service (NHLS) prices | Direct allocation |
| Non-HIV/Diabetes personnel | Managerial, cleaning and security staff | Facility utilisation | Facility expenditure | Facility headcount |
| Building operating and maintenance | Electricity, water, other utilities, phone, cleaning, transport, and stationery | Facility utilisation | Facility expenditure | Facility headcount |
| ***OTHER RELATED HEALTH CARE PROVIDERS*** | | | | |
| Public clinic visit | Average cost per visit | Patient interviews | Facility expenditure | Facility headcount |
| Public hospital ER/OPD visit | Average cost per OPD visit | Patient Interviews | Facility expenditure | Facility IP days and OP headcount |
| Public hospital Inpatient day | Average cost per inpatient day | Patient Interviews | Facility expenditure | Facility IP days and OP headcount |

**Table 1:** Measuring and valuing provider costs

For peer review only



For peer review only - http://bmjopen.bmj.com/site/about/guidelines.xhtml

***Measurement of resource use***

For the intervention costs an ingredients approach will be used to estimate resources. Routine data linked to intervention protocols will be collected and used to assess resources directly consumed in the provision of the intervention, including supplies, manuals, and patient education materials. Facility observations of intervention delivery will be conducted for the economist to understand the intervention and the facility context; these observations will complement intervention cost data collection. The costs of intervention focused staff training and facility-level institutional strengthening and capacity development through organisational readiness workshops [41] will be included in the cost analysis in order to capture the full economic costs of integrating the intervention into the primary care service for chronic disease patients.

A time and motion tool (TMT) developed for this evaluation will measure counsellor time usage in delivering the intervention for both active arms in the study. Time tools have been used in analysis of human resource requirements for expanding access to HIV [42] and TB care [43] using lay health workers and have proved useful in highlighting the scope of duties of this cadre. To our knowledge there is limited evidence in the South African context on time costs of CHW-delivered mental health counselling. The TMT will enable an analysis of differences in counsellor time usage in the different intervention modalities, indicating opportunities for scale and scope efficiencies. It is anticipated that the costs associated with the TAU option will be minimal and any referral costs will be captured through patient self-report of services they have used (see next paragraph).

For the HIV and Diabetes services, a step-down methodology will be used to establish resource use. Staff time will be determined through review of facility organograms and interviews with senior managers. In addition, utilisation of HIV and Diabetes medicine and diagnostics will be estimated by applying protocol-based facility utilisation statistics.

Establishing the use of referral services is challenging given that there is no health information system that enables patient pathways through care to be established. Therefore, in order to establish patient utilisation of health services beyond their HIV and Diabetes care at the primary level, we will administer a patient questionnaire developed for the study at baseline, and at the 6 and 12 month assessments. Using this questionnaire we will capture health service utilisation for other primary care services (e.g. TB treatment), hospital services, emergency department care, paramedic care and referrals to mental health providers. Patients will be asked to quantify the number of these additional visits, and lengths of stay in hospital. Given concerns with patient recall, we have asked patients to estimate their usage of ambulatory services over the previous month and have used a recall period of 6 months for inpatient services. These data will then be extrapolated to estimate costs over the time horizon.

***Valuation of costs***

Once the utilisation of services has been established, we will estimate the unit cost of each service. This involves sourcing a value for each item (e.g. annual cost of employment multiplied by proportion of time spent), as well as an allocation factor (e.g. annual facility head count) allowing the estimation of a unit cost (cost of employment X proportion of time / headcount). These sources of data and allocation factors are outlined in detail in **Table 1**.

For capital items, costs will be estimated using the replacement value method [37]. In essence, the current replacement value of each items is calculated and annuitized using data on the estimated useful life of the item and an interest rate representing the opportunity cost component for capital, in order to estimate an equivalent annual cost. An estimated useful life of between 5-20 years for furniture and buildings will be applied as while an interest rate of 8% will be used as the annuitization factor based on the rate of return on government bonds in South Africa [37].



For peer review only - http://bmjopen.bmj.com/site/about/guidelines.xhtml

## Estimating costs for the patient perspective

### *Measurement of resource use*

For the patient perspective, we will use a questionnaire at baseline, 6 and 12 months to estimate resource use. This will capture any user fees associated with public, private or NGO-provided health services as well as traditional and faith-based therapies. In addition, we will ask patients to estimate their time spent accessing health care services, as well as out of pocket payments for transport, subsistence and accommodation. Travel, subsistence and accommodation costs will relate to both the patient and their caregiver, if the patient is accompanied by a caregiver to any of these services.

### *Valuation of costs*

Travel, subsistence and accommodation costs will be estimated using the out of pocket payments reported by patients. Time spent receiving health care will be valued using the equality of wages approach [44], [45]. We will apply this method as public health services in South Africa are typically accessed by those who have a lower socio-economic status. Using this technique, the time costs for all patients are valued at the average income reported by the survey population[44]. This approach is expected to provide a relatively accurate reflection of the opportunity cost of unpaid labour and is favoured over the minimum wage approach in this context as a large proportion of the patients are unemployed resulting in a possible overestimate of the value of the time loss if the minimum wage is used [44]. In addition, we will ask patients if they have lost any income through seeking health care.

**Figure 2** summarises the intervention arms, costs, and outcomes that will be included in this study. All costs will be valued at 2018/2019 prices and, to allow comparability with other studies will be converted from South African Rand (ZAR) to United States Dollars (US$), using appropriate conversion rates for the same period obtained from OANDA, an online currency exchange rate conversion site. Costs incurred during earlier periods (such as manual development) will be inflated using the Consumer Price Index to reflect 2018/2019 prices.

ew only



For peer review only - http://bmjopen.bmj.com/site/about/guidelines.xhtml

## Analyses

### *Statistical analysis of costs and effects*

Mean provider costs and patient costs, will be calculated from baseline until the end of the intervention period. In addition, we will explore the extrapolation of costs to later periods if data allow for this. Cost differences between the MIND-TAU, and the MIND-DES and MIND-DED alternatives will be calculated. Sampling uncertainty for cost data will be estimated using statistical methods that account for the non-normal distribution of costs. Effect in terms of CES-D, AUDIT, HIV viral load, HBA1c, and the QALY will be analysed using linear regression on intervention type.

### *Determining cost-effectiveness*

The analysis will be conducted in line with the CHEERS (Consolidated Health Economic Evaluation Reporting Standards) guidelines [46]. Decision analytical models [37] integrating the cost and outcomes of the different intervention alternatives will be developed to assess cost-effectiveness. As a meaningful impact of the MIND-DES and MIND-DED on both costs and effects is anticipated, ICERs will be estimated in terms of incremental cost per natural unit and/or intermediate outcome for the cost effectiveness analysis (CEA), and incremental cost per QALY gained for the cost utility analysis (CUA). As the trial is focused on health systems strengthening for the delivery of mental health services, the decision rule applies to maximising health within the health care budget constraint. Therefore ICERs will be calculated from the provider perspective and , the cost per QALY gained will be compared to the cost-effectiveness thresholds (CETs) for LMIC settings [47]. If our intervention's ICER is less than the chosen CET this will mean that diverting resources to the intervention will increase population health, and if the ICER is more than the CET the intervention is not cost effective. Patient costs will be collected and reported on separately. In addition, to satisfy the requirements of common guidelines [46] and to allow for some degree of comparability with other studies, the ICERs will also be presented for the societal perspective, i.e. including both provider and patient costs.

### *Sensitivity analyses*

The decision analytical models will allow for univariate and multivariate sensitivity analyses to be conducted. This will enable the robustness of study findings to be tested by varying key parameters such as utilisation rates and assessing the impact on cost-effectiveness results. Standard areas of analytical focus will be considered, including testing sensitivity around the resource cost and prices, and theoretical controversies [48] for example: TTO weights [49] and discounting of outcomes [36]. Further analyses to inform the economics of task-sharing will include sensitivity analysis focused on economies and diseconomies of scale and scope, using detailed data from the TMT [50]. This will include testing variations in staff hours worked, variations in staff mix, lay counsellor funding models, and infrastructure investments. The impact of intervention dosage on outcomes will also be assessed in the sensitivity analyses. Statistical methods including multiple imputation will be used to manage the effect of missing information on costs and effects [51]. These sensitivity analyses are summarised in **Table 2**. Probabilistic sensitivity analysis (which allows application of confidence intervals to point estimates) will be used for sensitivity analyses of all relevant individual level variables. The sensitivity analyses will be used to produce a cost-effectiveness acceptability curve (CEAC).

| **Simple sensitivity analysis** | **Base-Case** | **Range** |
|---|---|---|
| Discount rate | 3% | 0%-10% |
| QALYs: HRQoL weights | EQ-5D valued using UK TTO | EQ-5D valued using Zimbabwe TTO |
| Unit costs | Primary data and data from published sources and official statistics for South Africa | Varied within plausible ranges as determined from a literature review of South African cost studies |
| Missing data | Multiple imputation | Complete case analysis |
| **Probabilistic sensitivity analysis** | | |



For peer review only - http://bmjopen.bmj.com/site/about/guidelines.xhtml

| | | |
|---|---|---|
| Intervention utilization data | Mean value | 95% uncertainty interval |
| Referral service utilization data | Mean value | 95% uncertainty interval |
| EQ-5D/CESD/AUDIT data | Mean value | 95% uncertainty interval |
| Clinical outcomes (HbA1c, HIV-1 RNA) | Mean value | 95% uncertainty interval |

**Table 2:** Planned Sensitivity Analyses

***Budget Impact Analysis***

Trial based estimates of intervention uptake and population based estimates of number in need will be used will be used in budget impact approximations which will be conducted using a mathematical programming approach [52], [53]. These approximations will inform discussions on the fiscal implications of investing in primary care mental health services [54].

**ETHICS AND DISSEMINATION**

In the CEA we foresee minimal risks to participants, as patient level data will be collected through the main trial, where, in order to minimise the risk of improper disclosure of information study staff will be required to sign a staff confidentiality agreement, and be trained and certified in protecting human participants. Data collection for the trial will be conducted using CAPI in which only a unique study identified is used. Knowledge translation will be through policy briefs, social media and as well as peer-reviewed papers, and presentations at health economics and mental health policy conferences. Engagement with department of health policy makers and other key stakeholders will be through a policy dialogue workshop linked to the main trial's Stake Holder Advisory Group (SAG). Dissemination of results to the public will be through press briefings in national and local media.

**DISCUSSION**

The delivery of psychotherapy using varying models of task-sharing to less specialised health workers in primary care has been shown to be cost-effective in HIC settings across a range of mental health conditions [55]–[57]but has limited empirical economic evidence in LMICs [50], [58], [59]. This study will provide evidence to address this gap. Through the cost-effectiveness analysis, the relative efficiency of each delivery mechanism will be established, enabling a discussion around which model produces the best outcomes given the available resources. As staff time is expected to be one of the main drivers of costs due to the nature of the intervention, we have developed a time tool that will allow us to directly measure staff time usage and investigate any potential excess capacity (hypothesized to occur with the MIND-DED counsellors), and scope economies (hypothesized to occur with the MIND-DES counsellors) which would impact the comparative efficiency of the different alternatives and thus the cost effectiveness results, as suggested in a similar study [50]. The cost-utility analysis will aid health service planners in their decision making around resource allocation and may be useful for guiding priority setting decisions both within the mental health budget space and across competing health priorities. To further support policy maker decisions, the affordability of implementing the intervention will need to be assessed, this will require further analysis using trial based evidence on intervention uptake and estimates of population level numbers in need to approximate the budget impact of equitably implementing the service [53].

Policy discussions on the integration of services to provide holistic care for patients requires economic evidence to support implementation in resource-limited contexts [4], [23]. By estimating the full economic costs of integrating the mental health treatment into the primary care offering for patients with chronic physical diseases, this study will provide much needed economic evidence to support this policy narrative [4], [60], [61]. Empirical data on the costs of upskilling and training staff to deliver the intervention and the time costs of the alternative human resourcing strategies will also aid in understanding how this cadre of staff organise their work and may highlight opportunities for efficiency gains. This is timely in the current policy window where the idea of a "facility counsellor" is



For peer review only - http://bmjopen.bmj.com/site/about/guidelines.xhtml

being discussed, whose role may span a range of counselling duties including: adherence counselling for TB/HIV, chronic non-communicable physical diseases and mental health counselling. The integration of services is a key health systems focus [62] requiring economic evidence [23]. The integration of services at the primary care level [63] to manage communicable and non-communicable chronicity is a global challenge [20], [22], [64], [65] and a key strategic aim of the Department of Health [66] for expanding access to mental health services and managing chronic diseases in South Africa [27], [66]. Brief interventions for depression and alcohol use disorders lack a common outcome measure for use in cost-effectiveness analysis [67] thus limiting comparison across interventions [68]. This study will provide unique cost-effectiveness evidence using the QALY as an outcome measure for such interventions in the South African context [50]. This will allow comparability of cost effectiveness results [69] and inform value for money decision making [47], [70]. This is of particular importance for South Africa as discussions around cost-effectiveness thresholds [70] are being increasingly presented in the health policy space in the prelude to National Health Insurance. This protocol also responds to the call to publish health economics protocols alongside trial protocols to reduce publication bias of RCT results [71].

Potential challenges to this study include recall bias due to the reliance on patient-reported health service use for our estimations of service utilisation. To limit recall bias, a one month recall period will be used for ambulatory services and 6 month recall for hospitalisations [51]. Another possible limitation is that only a few patients may be referred for mental health care for CMD in the TAU option so we may not fully capture referral costs thus potentially underestimating cost-effectiveness. Another potential limitation is that the short follow-up period of 12 months may not capture the extent of the benefit that patients and the health system could gain through the availability of this service. For example, the degree to which the socio-economic benefits and the returns to mental health associated with such interventions [72] may not be fully evident by the 12 month end-point. This is a common problem associated with trial-based economic evaluations [73].

**FUTURE WORK**

Further work associated with this economic evaluation includes the use of modelling techniques to estimate the long-term benefits of the intervention both at the patient and system level and applying cost-benefit techniques to strengthen the evidence base for the mental health investment case.

**CONCLUSION**

Priority setting and decisions around the scale up of an integrated comprehensive primary care service need to be informed by micro- and macro-level economic analyses of the investment requirements, impact, and equity implications of delivering key services such as counselling. It is anticipated that the cost estimates, the CEA and budget impact analysis of this intervention will be a useful contribution to priority setting and policy making during the current reconfiguration of South Africa's health system [74].It is hoped that the economic evaluation of project MIND will provide much needed evidence on the cost-effectiveness of integrating mental health services into primary care that could support a business case for investing in these services.

**PATIENT AND PUBLIC INVOLVEMENT**

Patients and the public were engaged with through the trial, and were not directly involved in this study

**FUNDING STATEMENT**

This work is supported by the joint funded initiatives of the British Medical Research Council, Wellcome Trust and DFID (MR/M014290/1). BM is supported by the South African Medical Research Council.



For peer review only - http://bmjopen.bmj.com/site/about/guidelines.xhtml

**COMPETING INTERESTS STATEMENT**

The authors declare that they have no competing interests.

**AUTHOR CONTRIBUTIONS**

VM-M, SC and BM made substantial contributions to the conception and design of the study. The first draft of the manuscript was written by VM-M and critically revised by all authors (VM-M, BM,KS,CL,TL ,SC) for important intellectual content. All authors read and approved the final manuscript.

**ABBREVIATIONS**

ART: Antiretroviral therapy; AUDIT: Alcohol Use Disorders Identification Test; CEA: Cost Effectiveness Analysis; CES-D: Center for Epidemiology Scale on Depression; CET: Cost Effectiveness Threshold; CHW: Community health worker; CMD: Common mental disorder; CUA: Cost Utility Analysis; HIC: High Income Country; HRQoL: Health-related quality of life; LMIC: Low- and middle-income country; MI: Motivational interviewing; NCD: Non-communicable disease; PHC: Primary health care; PST: Problem-solving therapy; QALY: Quality-adjusted life year; SAG: Stakeholder Advisory Group; TMT: Time and motion tool; TAU: Treatment as usual; WCDOH: Western Cape Department of Health

**ACKNOWLEDGEMENTS**

We thank all WCDOH facilities, which have agreed to participate in the trial, and our Stakeholder Advisory Group for their contributions.

For peer review only



For peer review only - http://bmjopen.bmj.com/site/about/guidelines.xhtml

**REFERENCES**

[1] V. Patel *et al.*, “The Lancet Commission on global mental health and sustainable development,” *Lancet*, vol. 392, no. 10157, pp. 1553–1598, 2018.

[2] H. A. Whiteford *et al.*, “Global burden of disease attributable to mental and substance use disorders: findings from the Global Burden of Disease Study 2010,” *Lancet*, vol. 382, no. 9904, pp. 1575–1586, 2013.

[3] S. Moussavi, S. Chatterji, E. Verdes, A. Tandon, V. Patel, and B. Ustun, “Depression , chronic diseases , and decrements in health : results from the World Health Surveys,” *Lancet*, pp. 851–858, 2007.

[4] V. K. Ngo *et al.*, “Grand Challenges: Integrating Mental Health Care into the Non-Communicable Disease Agenda,” *PLoS Med.*, vol. 10, no. 5, pp. 1–6, 2013.

[5] D. Vancampfort *et al.*, “The prevalence of diabetes mellitus type 2 in people with alcohol use disorders: a systematic review and large scale meta-analysis,” *Psychiatry Res.*, vol. 246, no. October, pp. 394–400, 2016.

[6] K. Sorsdahl, R. Sewpaul, M. Evans, P. Naidoo, B. Myers, and D. J. Stein, “The association between psychological distress, alcohol use and physical non-communicable diseases in a nationally representative sample of South Africans,” *J. Health Psychol.*, vol. 23, no. 4, pp. 618–628, 2018.

[7] M. G. Neuman, M. Schneider, R. M. Nanau, and C. Parry, “Alcohol consumption, progression of disease and other comorbidities, and responses to antiretroviral medication in people living with HIV,” *AIDS Res. Treat.*, vol. 2012, 2012.

[8] C. Bernard, F. Dabis, and N. De Rekeneire, “Prevalence and factors associated with depression in people living with HIV in sub-Saharan Africa: A systematic review and meta-analysis,” *PLoS One*, vol. 12, no. 8, pp. 1–22, 2017.

[9] L. E. Egede, D. Zheng, and K. Simpson, “Comorbid depression is associated with increased health care use and expenditures in individuals with diabetes,” *Diabetes Care*, vol. 25, no. 3, pp. 464–470, 2002.

[10] W. J. Katon, J. E. Russo, M. Von Korff, E. H. B. Lin, E. Ludman, and P. S. Ciechanowski, “Long-term effects on medical costs of improving depression outcomes in patients with depression and diabetes,” *Diabetes Care*, vol. 31, no. 6, pp. 1155–1159, 2008.

[11] B. M. Mayosi, J. E. Lawn, A. van Niekerk, D. Bradshaw, S. S. Abdool Karim, and H. M. Coovadia, “Health in South Africa: changes and challenges since 2009.,” *Lancet*, vol. 380, no. 9858, pp. 2029–43, Dec. 2012.

[12] D. J. Stein *et al.*, “Lifetime prevalence of psychiatric disorders in South Africa.,” *Br. J. Psychiatry*, vol. 192, pp. 112–117, 2008.

[13] B. D. Fulton, R. M. Scheffler, S. P. Sparkes, E. Y. Auh, M. Vujicic, and A. Soucat, “Health workforce skill mix and task shifting in low income countries: a review of recent evidence.,” *Hum. Resour. Health*, vol. 9, no. 1, p. 1, 2011.

[14] WHO, “Treat, Train, Retain: Task Shifting Global Recomendations and Guidelines,” *World Heal. Organ.*, p. 96, 2008.

[15] WHO, “mhGAP Intervention Guide for mental, neurological and substance use disorders in non-specialized health settings Version 2.0,” *mhGAP Newsl.*, vol. 1, no. 9, pp. 1–4, 2015.

[16] R. Joshi *et al.*, “Task Shifting for Non-Communicable Disease Management in Low and Middle



For peer review only - http://bmjopen.bmj.com/site/about/guidelines.xhtml

Income Countries – A Systematic Review," *PLoS One*, vol. 9, no. 8, p. e103754, 2014.

[17] D. Javadi, I. Feldhaus, A. Mancuso, and A. Ghaffar, "Applying systems thinking to task shifting for mental health using lay providers: a review of the evidence," *Glob. Ment. Heal.*, vol. 4, p. e14, 2017.

[18] T. A. Bruckner *et al.*, "The mental health workforce gap in low- and middle-income countries: A needs-based approach," *Bull. World Health Organ.*, vol. 89, no. 3, pp. 184–194, 2011.

[19] J. Mugisha, M. De Hert, B. Stubbs, D. Basangwa, and D. Vancampfort, "Physical health policies and metabolic screening in mental health care systems of Sub Saharan african Countries: a s systematic review," *Int. J. Ment. Health Syst.*, pp. 1–7, 2017.

[20] G. Thornicroft *et al.*, "Integrated care for people with long-term mental and physical health conditions in low-income and middle-income countries," *The Lancet Psychiatry*, vol. 0366, no. 18, pp. 1–13, 2018.

[21] J. E. Haberer *et al.*, "Improving antiretroviral therapy adherence in resource-limited settings at scale: A discussion of interventions and recommendations," *J. Int. AIDS Soc.*, vol. 20, no. 1, pp. 1–15, 2017.

[22] F. L. H. Chuah *et al.*, "Interventions and approaches to integrating HIV and mental health services: a systematic review," *Health Policy Plan.*, 2017.

[23] S. I. Watson, H. Sahota, C. A. Taylor, Y.-F. Chen, and R. J. Lilford, "Cost-effectiveness of health care service delivery interventions in low and middle income countries: a systematic review," *Glob. Heal. Res. Policy*, vol. 3, no. 17, 2018.

[24] B. Myers *et al.*, "Comparing dedicated and designated models of integrating mental health into chronic disease care : study protocol for a cluster randomized controlled trial," pp. 1–13, 2018.

[25] C. Lund, S. Kleintjes, R. Kakuma, and A. J. Flisher, "Public sector mental health systems in South Africa: Inter-provincial comparisons and policy implications," *Soc. Psychiatry Psychiatr. Epidemiol.*, vol. 45, no. 3, pp. 393–404, 2010.

[26] I. Petersen and C. Lund, "Mental health service delivery in South Africa from 2000 to 2010: One step forward, one step back," *South African Med. J.*, vol. 101, no. 10, pp. 751–757, 2011.

[27] National Department of Health Republic of South Africa, "National Mental Health Policy Framework and Strategic Plan 2013-2020," Pretoria, 2013.

[28] K. Sorsdahl *et al.*, "Adapting a blended motivational interviewing and problem-solving intervention to address risky substance use amongst South Africans.," *Psychother. Res.*, vol. 3307, no. April, pp. 37–41, 2014.

[29] B. Myers, D. J. Stein, B. Mtukushe, and K. Sorsdahl, "Feasibility and Acceptability of Screening and Brief Interventions to Address Alcohol and Other Drug Use among Patients Presenting for Emergency Services in Cape Town, South Africa.," *Adv. Prev. Med.*, vol. 2012, p. 569153, 2012.

[30] K. Sorsdahl *et al.*, "Screening and brief interventions for substance use in emergency departments in the Western Cape province of South Africa: Views of health care professionals," *Int. J. Inj. Contr. Saf. Promot.*, vol. 21, no. 3, pp. 236–243, 2014.

[31] K. Sorsdahl *et al.*, "The efficacy of a blended motivational interviewing and problem solving therapy intervention to reduce substance use among patients presenting for emergency services in South Africa: A randomized controlled trial," *Subst. Abus. Treat. Prev. Policy*, vol.



For peer review only - http://bmjopen.bmj.com/site/about/guidelines.xhtml

10, no. 1, pp. 1–10, 2015.

[32] N. T. Van Dam and M. Earleywine, "Validation of the Center for Epidemiologic Studies Depression Scale-Revised (CESD-R): Pragmatic depression assessment in the general population," *Psychiatry Res.*, vol. 186, no. 1, pp. 128–132, 2011.

[33] T. F. Babor, J. C. Higgins-Biddle, J. B. Saunders, and M. G. Monteiro, "The Alcohol Use Disorders Identification Test Guidelines for Use in Primary Care," *World Heal. Organ.*, pp. pp1-40, 2001.

[34] M. van Reenen and B. Janssen, "EQ-5D-5L User Guide - Basic information on how to use the EQ-5D-5L instrument.," *Version 2.1*, no. October, 2015.

[35] W. Hollingworth, D. McKell-Redwood, L. Hampson, and C. Metcalfe, "Cost-utility analysis conducted alongside randomized controlled trials: Are economic end points considered in sample size calculations and does it matter?," *Clin. Trials*, vol. 10, no. 1, pp. 43–53, 2013.

[36] A. E. Attema, W. B. F. Brouwer, and K. Claxton, "Discounting in Economic Evaluations," *Pharmacoeconomics*, vol. 36, no. 7, pp. 745–758, 2018.

[37] M. F. Drummond, M. J. Sculpher, K. Claxton, G. L. Stoddart, and G. W. Torrance, *Methods for the Economic Evaluation of Health Care Programmmes*, 4th ed. Oxford: Oxford University Press, 2015.

[38] J. Jelsma, S. Mkoka, L. Amosun, and J. Nieuwveldt, "The reliability and validity of the Xhosa version of the EQ-5D.," *Disabil. Rehabil.*, vol. 26, no. 2, pp. 103–108, 2004.

[39] R. Rabin and F. De Charro, "EQ-5D: A measure of health status from the EuroQol Group," *Ann. Med.*, vol. 33, no. 5, pp. 337–343, 2001.

[40] S. Kularatna, J. A. Whitty, N. W. Johnson, and P. A. Scuffham, "Health State Valuation in Low- and Middle-Income Countries: A Systematic Review of the Literature," *Value Heal.*, vol. 16, no. 6, pp. 1091–1099, 2013.

[41] C. Brooke-Sumner, K. Sorsdahl, C. Lombard, P. Petersen-Williams, and B. Myers, "Protocol for development and validation of a context-appropriate tool for assessing organisational readiness for change in primary health clinics in South Africa," *BMJ Open*, vol. 8, no. 4, p. e020539, 2018.

[42] H. Naburi *et al.*, "The potential of task-shifting in scaling up services for prevention of mother-to-child transmission of HIV: a time and motion study in Dar es Salaam, Tanzania.," *Hum. Resour. Health*, vol. 15, no. 1, p. 35, 2017.

[43] W. A. Odendaal and S. Lewin, "The provision of TB and HIV/AIDS treatment support by lay health workers in South Africa: a time-and-motion study.," *Hum. Resour. Health*, vol. 12, no. 1, p. 18, 2014.

[44] N. Foster, A. Vassall, S. Cleary, L. Cunnama, G. Churchyard, and E. Sinanovic, "The economic burden of TB diagnosis and treatment in South Africa," *Soc. Sci. Med.*, vol. 130, pp. 42–50, 2015.

[45] V. Mauch, N. Woods, B. Kirubi, H. Kipruto, J. Sitienei, and E. Klinkenberg, "Assessing access barriers to tuberculosis care with the tool to Estimate Patients' Costs: Pilot results from two districts in Kenya," *BMC Public Health*, vol. 11, pp. 18–21, 2011.

[46] D. Husereau *et al.*, "Consolidated Health Economic Evaluation Reporting Standards (CHEERS) statement," *Eur. J. Heal. Econ.*, vol. 14, no. 3, pp. 367–372, 2013.



For peer review only - http://bmjopen.bmj.com/site/about/guidelines.xhtml

[47] B. Woods, P. Revill, M. Sculpher, and K. Claxton, "Country-Level Cost-Effectiveness Thresholds: Initial Estimates and the Need for Further Research," *Value Heal.*, vol. 19, no. 8, pp. 929–935, 2016.

[48] A. M. Gray and T. Wilkinson, "Economic evaluation of healthcare interventions: Old and new directions," *Oxford Rev. Econ. Policy*, vol. 32, no. 1, pp. 102–121, 2016.

[49] A. Manca, N. Hawkins, and M. J. Sculpher, "Estimating mean QALYs in trial-based cost-effectiveness analysis: The importance of controlling for baseline utility," *Health Econ.*, vol. 14, no. 5, pp. 487–496, 2005.

[50] R. Dwommoh *et al.*, "Brief interventions to address substance use among patients presenting to emergency departments in resource poor settings : a cost ‑ effectiveness analysis," *Cost Eff. Resour. Alloc.*, pp. 1–11, 2018.

[51] D. Hughes *et al.*, "Conducting Economic Evaluations Alongside Randomised Trials: Current Methodological Issues and Novel Approaches," *Pharmacoeconomics*, vol. 34, no. 5, pp. 447–461, 2016.

[52] D. M. Epstein, Z. Chalabi, K. Claxton, and M. Sculpher, "Efficiency, equity, and budgetary policies: Informing decisions using mathematical programming," *Med. Decis. Mak.*, vol. 27, no. 2, pp. 128–137, 2007.

[53] S. Cleary, G. Mooney, and D. Mcintyre, "Equity and Efficiency in HIV - Treatment in South Africa: The Contribution of Mathematical Programming to Priority Setting.," *Health Econ.*, vol. 19, pp. 1166–1180, 2010.

[54] S. D. Sullivan *et al.*, "Budget impact analysis - Principles of good practice: Report of the ISPOR 2012 budget impact analysis good practice II task force," *Value Heal.*, vol. 17, no. 1, pp. 5–14, 2014.

[55] W. Katon *et al.*, "Cost-effectiveness of a multicondition collaborative care intervention," *Arch. Gen. Psychiatry*, vol. 69, no. 5, pp. 506–514, 2012.

[56] J. W. Hay, W. J. Katon, K. Ell, P.-J. Lee, and J. J. Guterman, "Cost-Effectiveness Analysis of Collaborative Care Management of Major Depression among Low-Income, Predominantly Hispanics with Diabetes," *Value Heal.*, vol. 15, no. 2, pp. 249–254, 2012.

[57] D. A. Richards *et al.*, "Clinical effectiveness of collaborative care for depression in UK primary care (CADET): cluster randomised controlled trial," *BMJ*, vol. 347, no. 14, p. f4913, 2013.

[58] V. Patel, D. Chisholm, S. Rabe-Hesketh, F. Dias-Saxena, G. Andrew, and A. Mann, "Efficacy and cost-effectiveness of drug and psychological treatments for common mental disorders in general health care in Goa, India: a randomised, controlled trial.," *Lancet (London, England)*, vol. 361, no. 9351, pp. 33–9, 2003.

[59] D. Siskind, F. K. Baingana, and J. Kim, "Cost-Effectiveness of Group Psycotherapy Depression For Depression in Uganda," *J. Ment. Heal. Policy Econ.*, vol. 11, pp. 127–133, 2008.

[60] I. Petersen, C. Lund, A. Bhana, and A. J. Flisher, "A task shifting approach to primary mental health care for adults in South Africa: Human resource requirements and costs for rural settings," *Health Policy Plan.*, vol. 27, no. 1, pp. 42–51, 2012.

[61] H. Jack *et al.*, "Closing the mental health treatment gap in South Africa: A review of costs and cost-effectiveness," *Glob. Health Action*, vol. 7, no. SUPP.1, pp. 1–11, 2014.

[62] R. Atun, T. De Jongh, F. Secci, K. Ohiri, and O. Adeyi, "Integration of targeted health interventions into health systems: A conceptual framework for analysis," *Health Policy Plan.*,



For peer review only - http://bmjopen.bmj.com/site/about/guidelines.xhtml

vol. 25, no. 2, pp. 104–111, 2010.

[63] L. Dudley and P. Garner, "Strategies for integrating primary health services in low- and middle-income countries at the point of delivery.," *Cochrane Database Syst. Rev.*, no. 2, p. CD003318, 2011.

[64] V. Haldane *et al.*, "Integrating cardiovascular diseases, hypertension, and diabetes with HIV services: a systematic review," *AIDS Care - Psychol. Socio-Medical Asp. AIDS/HIV*, vol. 30, no. 1, pp. 103–115, 2018.

[65] C. G. Kemp *et al.*, "Implementation science for integration of HIV and non-communicable disease services in sub-Saharan Africa: A systematic review," *Aids*, vol. 32, no. May, pp. S93–S105, 2018.

[66] Western Cape Government, "Healthcare 2030: The Road to Wellness," Cape Town, 2014.

[67] A. J. Cowell, J. M. Brown, M. J. Mills, R. H. Bender, and B. J. Wedehase, "Cost-effectiveness analysis of motivational interviewing with feedback to reduce drinking among a sample of college students," *J. Stud. Alcohol Drugs*, vol. 73, no. 2, pp. 226–37, 2012.

[68] A. J. Cowell, J. W. Bray, M. J. Mills, and J. M. Hinde, "Conducting economic evaluations of screening and brief intervention for hazardous drinking: Methods and evidence to date for informing policy," *Drug Alcohol Rev.*, vol. 29, no. 6, pp. 623–630, 2010.

[69] M. C. Weinstein, G. Torrance, and A. Mcguire, "QALYs : The Basics," vol. 12, 2009.

[70] P. Thokala, J. Ochalek, A. A. Leech, and T. Tong, "Cost-Effectiveness Thresholds: the Past, the Present and the Future," *Pharmacoeconomics*, pp. 1–14, 2018.

[71] T. Powell-Jackson *et al.*, "Trials and tribulations: cross-learning from the practices of epidemiologists and economists in the evaluation of public health interventions," *Health Policy Plan.*, no. May, pp. 1–5, 2018.

[72] C. Lund *et al.*, "Poverty and common mental disorders in low and middle income countries: A systematic review.," *Soc. Sci. Med.*, vol. 71, no. 3, pp. 517–528, 2010.

[73] H. A. Glick, J. A. Doshi, S. S. Sonnad, and D. Polsky, *Economic Evauation in Clinical Trials*, 2nd ed. New York: Oxford University Press, 2015.

[74] L. Rispel, J. Moorman, M. Chersich, J. Goudge, N. Nxumalo, and T. Ndou, "REVITALISING PRIMARY HEALTH CARE IN SOUTH AFRICA: Review of primary health care package, norms and standards," Johannesburg, 2010.

**Figure 1** Economic Evaluation: Analyses, Outcomes, Measurement and Assessment Timing

**Figure 2** Project MIND: Intervention Arms, Costs, Outcomes & Economic Evaluation

**Table 1:** Measuring and valuing provider costs

**Table 2:** Planned Sensitivity Analyses



For peer review only - http://bmjopen.bmj.com/site/about/guidelines.xhtml

| *Analysis* | *Assessment/ Economic Evaluation Outcomes* | **Measurement instrument OR Clinical Parameter** | **Assessment Timing** (Trial recruitment: starts 2017- ends 2019) | | | |
|---|---|---|---|---|---|---|
| | | Intervention start | Baseline | 6 months | 1 Year | Intervention assessment end |
| | ***1º Outcomes*** | | | | | |
| CEA | Harmful alcohol use (Self-report interview) | AUDIT | ✓ | ✓ | ✓ | |
| CEA | Depression (Self-report interview) | CES-D | ✓ | ✓ | ✓ | |
| CEA | ***2º Outcomes*** | | | | | |
| CEA | Diabetes treatment failure risk | HbA1c | ✓ | ✓ | ✓ | |
| CEA | | BMI | | | | |
| CEA | HIV treatment failure risk | HIV-1 RNA (HIV-viral load) | ✓ | ✓ | ✓ | |
| CEA | Chronic Disease Medication Adherence | Visual analogue Scale and CASE adherence Index | ✓ | ✓ | ✓ | |
| CUA | ***Multi-attribute Outcomes*** | | | | | |
| CUA | QALY | EQ-5D | ✓ | ✓ | ✓ | |

For peer review only

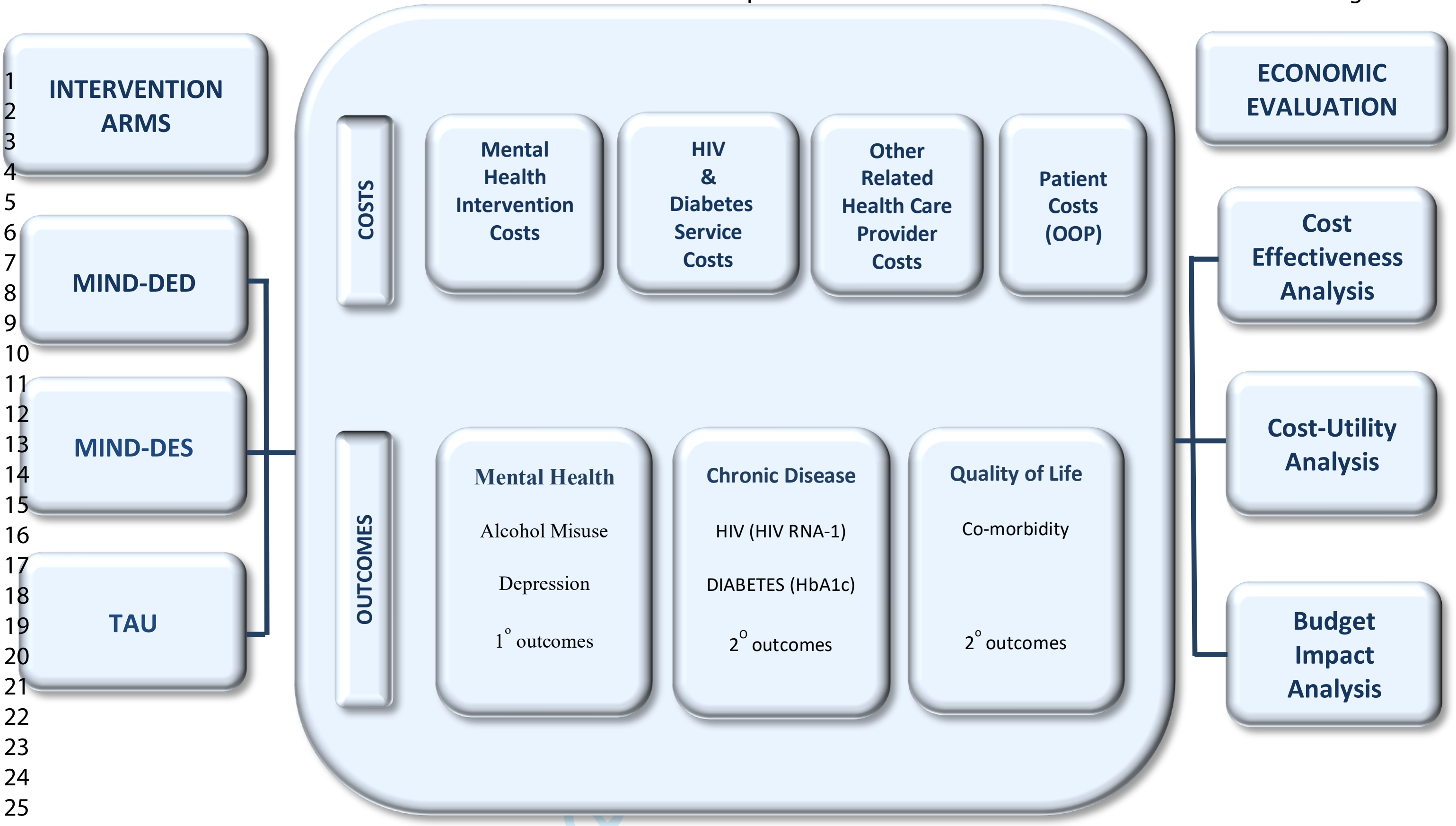
INTERVENTION ARMS
MIND-DED
MIND-DES
TAU
COSTS
Mental Health Intervention Costs
HIV & Diabetes Service Costs
Other Related Health Care Provider Costs
Patient Costs (OOP)
OUTCOMES
Mental Health
Alcohol Misuse
Depression
1° outcomes
Chronic Disease
HIV (HIV RNA-1)
DIABETES (HbA1c)
2° outcomes
Quality of Life
Co-morbidity
2° outcomes
ECONOMIC EVALUATION
Cost Effectiveness Analysis
Cost-Utility Analysis
Budget Impact Analysis

For peer review only

**CHEERS checklist**
**Items to include when reporting economic evaluations of health interventions**

Further discussion of the checklist may be accessed from the ISPOR Health Economic Evaluation Publication Guidelines – CHEERS: Good Reporting Practices webpage: http://www.ispor.org/TaskForces/EconomicPubGuidelines.asp.

| Section /item | Item No | Recommendation | Reported on page No/ line No |
|---|---|---|---|
| **Title and abstract** | | | |
| Title | 1 | Identify the study as an economic evaluation or use more specific terms such as "cost-effectiveness analysis", and describe the interventions compared. | Page 1 |
| Abstract | 2 | Provide a structured summary of objectives, perspective, setting, methods (including study design and inputs), results (including base case and uncertainty analyses), and conclusions. | Page 2 |
| **Introduction** | | | |
| Background and objectives | 3 | Provide an explicit statement of the broader context for the study. | Page 3 |
| | | Present the study question and its relevance for health policy or practice decisions. | Page 5 |
| **Methods** | | | |
| Target population and subgroups | 4 | Describe characteristics of the base case population and subgroups analysed, including why they were chosen. | Page 5 |
| Setting and location | 5 | State relevant aspects of the system(s) in which the decision(s) need(s) to be made. | Page 4 |
| Study perspective | 6 | Describe the perspective of the study and relate this to the costs being evaluated. | Page 5,6,7 |
| Comparators | 7 | Describe the interventions or strategies being compared and state why they were chosen. | Page 4 |
| Time horizon | 8 | State the time horizon(s) over which costs and consequences are being evaluated and say why appropriate. | Page 5 |
| Discount rate | 9 | Report the choice of discount rate(s) used for costs and outcomes and say why appropriate. | Page 5 |
| Choice of health outcomes | 10 | Describe what outcomes were used as the measure(s) of benefit in the evaluation and their relevance for the type of analysis performed. | Page 4 |
| Measurement of effectiveness | 11a | *Single study-based estimates:* Describe fully the design features of the single effectiveness study and why the single study was a sufficient source of clinical effectiveness data. | Page 6 |
| | 11b | *Synthesis-based estimates*: Describe fully the methods used for identification of included studies and synthesis of clinical effectiveness data. | |
| Measurement and valuation of preference based outcomes | 12 | If applicable, describe the population and methods used to elicit preferences for outcomes. | Page 6 |
| Estimating resources and costs | 13a | *Single study-based economic evaluation:* Describe approaches used to estimate resource | Page 6,7 |

For peer review only - http://bmjopen.bmj.com/site/about/guidelines.xhtml

| Section /item | Item No | Recommendation | Reported on page No/ line No |
|---|---|---|---|
| | | use associated with the alternative interventions. Describe primary or secondary research methods for valuing each resource item in terms of its unit cost. Describe any adjustments made to approximate to opportunity costs. | |
| | 13b | *Model-based economic evaluation:* Describe approaches and data sources used to estimate resource use associated with model health states. Describe primary or secondary research methods for valuing each resource item in terms of its unit cost. Describe any adjustments made to approximate to opportunity costs. | Not applicable |
| Currency, price date, and conversion | 14 | Report the dates of the estimated resource quantities and unit costs. Describe methods for adjusting estimated unit costs to the year of reported costs if necessary. Describe methods for converting costs into a common currency base and the exchange rate. | Page 8 |
| Choice of model | 15 | Describe and give reasons for the specific type of decision-analytical model used. Providing a figure to show model structure is strongly recommended. | Protocol – not applicable |
| Assumptions | 16 | Describe all structural or other assumptions underpinning the decision-analytical model. | Protocol – not applicable |
| Analytical methods | 17 | Describe all analytical methods supporting the evaluation. This could include methods for dealing with skewed, missing, or censored data; extrapolation methods; methods for pooling data; approaches to validate or make adjustments (such as half cycle corrections) to a model; and methods for handling population heterogeneity and uncertainty. | Page 9 |
| **Results** | | | |
| Study parameters | 18 | Report the values, ranges, references, and, if used, probability distributions for all parameters. Report reasons or sources for distributions used to represent uncertainty where appropriate. Providing a table to show the input values is strongly recommended. | Protocol – not applicable |
| Incremental costs and outcomes | 19 | For each intervention, report mean values for the main categories of estimated costs and outcomes of interest, as well as mean differences between the comparator groups. If applicable, report incremental cost-effectiveness ratios. | Protocol – not applicable |
| Characterising uncertainty | 20a | *Single study-based economic evaluation:* Describe the effects of sampling uncertainty for the estimated incremental cost and incremental effectiveness parameters, together with the impact of methodological assumptions (such as discount rate, study perspective). | Protocol – not applicable |
| | 20b | *Model-based economic evaluation:* Describe the effects on the results of uncertainty for all input parameters, and uncertainty related to the | Not applicable |

For peer review only

| Section /item | Item No | Recommendation | Reported on page No/ line No |
|---|---|---|---|
| | | structure of the model and assumptions. | |
| Characterising heterogeneity | 21 | If applicable, report differences in costs, outcomes, or cost-effectiveness that can be explained by variations between subgroups of patients with different baseline characteristics or other observed variability in effects that are not reducible by more information. | Protocol – not applicable |
| **Discussion** | | | |
| Study findings, limitations, generalisability, and current knowledge | 22 | Summarise key study findings and describe how they support the conclusions reached. Discuss limitations and the generalisability of the findings and how the findings fit with current knowledge. | Protocol discussion |
| **Other** | | | |
| Source of funding | 23 | Describe how the study was funded and the role of the funder in the identification, design, conduct, and reporting of the analysis. Describe other non-monetary sources of support. | Page 11 |
| Conflicts of interest | 24 | Describe any potential for conflict of interest of study contributors in accordance with journal policy. In the absence of a journal policy, we recommend authors comply with International Committee of Medical Journal Editors recommendations. | Information provided via the submission system |

For consistency, the CHEERS statement checklist format is based on the format of the CONSORT statement checklist

The citation for the CHEERS Task Force Report is: Husereau D, Drummond M, Petrou S, et al. Consolidated health economic evaluation reporting standards (CHEERS)—Explanation and elaboration: A report of the ISPOR health economic evaluations publication guidelines good reporting practices task force. Value Health 2013;16:231-50.

For peer review only

BMJ Open

# Integrating a Brief Mental Health Intervention into Primary Care Services for HIV and Diabetes Patients in South Africa: Study Protocol for a Trial Based Economic Evaluation.

| | |
|---|---|
| Journal: | *BMJ Open* |
| Manuscript ID | bmjopen-2018-026973.R2 |
| Article Type: | Protocol |
| Date Submitted by the Author: | 11-Mar-2019 |
| Complete List of Authors: | Mutyambizi-Mafunda, Vimbayi; University of Cape Town School of Public Health and Family Medicine, Health Economics Unit;<br>Myers, Bronwyn; South African Medical Research Council, Alcohol and Drug Abuse Research Unit<br>Sorsdahl, Katherine; University of Cape Town, Department of Psychiatry and Mental Health, Alan J Flisher Centre for Public Mental Health<br>Lund, Crick; University of Cape Town, Department of Psychiatry and Mental Health, Alan J Flisher Centre for Public Mental Health; Kings College London, Institute of Psychiatry, Psychology and Neuroscience, Health Services and Population Research<br>Naledi, Tracey; University of Cape Town School of Public Health and Family Medicine, Desmond Tutu HIV Research Centre; Western Cape Department of Health<br>Cleary, S; University of Cape Town School of Public Health and Family Medicine, Health Economics Unit |
| <b>Primary Subject Heading</b>: | Health economics |
| Secondary Subject Heading: | Mental health |
| Keywords: | cost-effectiveness analysis, common mental disorders, HIV, Diabetes, integrated treatment |
| | |

SCHOLARONE™
Manuscripts

# Integrating a Brief Mental Health Intervention into Primary Care Services for HIV and Diabetes Patients in South Africa: Study Protocol for a Trial Based Economic Evaluation.

**Authors:** Vimbayi Mutyambizi-Mafunda[1], Bronwyn Myers[2,3], Katherine Sorsdahl[4], Crick Lund[4,5], Tracey Naledi[6], Susan Cleary[1]

[1] Health Economics Unit, School of Public Health and Family Medicine, University of Cape Town, Cape Town, South Africa.

[2] Alcohol, Tobacco and Other Drug Research Unit, South African Medical Research Council, Francie van Zyl Drive, Tygerberg, Cape Town, 7505, South Africa

[3] Division of Addiction Psychiatry, Psychiatry and Mental Health, University of Cape Town, Cape Town, South Africa.

[4]Alan J Flisher Centre for Public Mental Health, Department of Psychiatry and Mental Health, University of Cape Town, Cape Town, South Africa.

[5] Centre for Global Mental Health, Institute of Psychiatry, Psychology and Neuroscience, King's College, London, UK.

[6] Western Cape Department of Health, 8 Riebeeck Street, Cape Town, South Africa.

**Author Email addresses:**

Vimbayi Mutyambizi-Mafunda - vimbayi.mafunda@uct.ac.za

Bronwyn Myers – Bronwyn.Myers@mrc.ac.za

Katherine Sorsdahl – katherine.sorsdahl@uct.ac.za

Crick Lund – crick.lund@uct.ac.za

Tracey Naledi – Tracey.Naledi@hiv-research.org.za

Susan Cleary – susan.cleary@uct.ac.za

**Corresponding Author**

**Name:** Vimbayi Mutyambizi-Mafunda

**Postal Address:** Health Economics Unit, University of Cape Town, School of Public Health & Family Medicine, Health Sciences Faculty, Anzio Road, Observatory, Cape Town,7925, South Africa.

**Email address:** vimbayi.mafunda@uct.ac.za

**Telephone Number:** +27 21 406 6558

**Word count:** excluding abstract and article summary - 4835



For peer review only - http://bmjopen.bmj.com/site/about/guidelines.xhtml

## ABSTRACT

**Introduction:** Depression and alcohol use disorders are international public health priorities for which there is a substantial treatment gap. Brief mental health interventions delivered by lay health workers in primary care services may reduce this gap. There is limited economic evidence assessing the cost-effectiveness of such interventions in low-and middle-income countries (LMIC). This paper describes the proposed economic evaluation of a health systems intervention testing the effectiveness, cost-effectiveness and cost-utility of two task-sharing approaches to integrating services for common mental disorders with HIV and Diabetes primary care services.

**Methods and analysis:** This evaluation will be conducted as part of a three-armed cluster-randomised controlled trial of clinical effectiveness. Trial clinical outcome measures will include primary outcomes for risk of depression and alcohol use, and secondary outcomes for risk of chronic disease (HIV and Diabetes) treatment failure. The cost-effectiveness analysis will evaluate cost per unit change in AUDIT and CES-D scores as well as cost per unit change in HIV RNA viral load and HBA1c, producing results of provider and patient cost per patient year for each study arm and chronic disease. The cost utility analyses will provide results of cost per quality adjusted life year (QALY) gained. Additional analyses relevant for implementation including budget impact analyses will be conducted to inform the development of a business case for scaling up the country's investment in mental health services.

**Ethics and dissemination:** The Western Cape Department of Health (WCDoH) (WC2016_RP6_9), the South African Medical Research Council (EC 004-2/2015), the University of Cape Town (089/2015), and Oxford University (OxTREC 2-17) provided ethical approval for this study. Results dissemination will include policy briefs, social media, peer-reviewed papers, a policy dialogue workshop and press briefings.

**Trial registration**: Pan African Clinical Trials registry (registration number PACTR201610001825405).

**Keywords:** cost-effectiveness analysis, common mental disorders, HIV, Diabetes, integrated treatment

## ARTICLE SUMMARY

### Strengths and limitations of this study

- This study will provide some of the first empirical evidence of the cost-effectiveness of integrating services for common mental disorders in the primary care offering for HIV and Diabetes.
- The study will generate findings that may guide priority setting through application of QALY based outcomes and decision-making around the implementation of mental health counselling within the health system.
- This paper adds to the growing body of protocols for economic evaluations conducted alongside RCTs.
- As health service use is self-reported, there is a possibility for recall bias, to limit this, a one month recall period will be used for ambulatory services and a 6 month recall for hospitalisation, and hospitalisations will be validated through the Department of Health Data Centre.
- The one year follow up period may reflect outcomes that do not fully represent the full benefit that patients and the health system could gain through the availability of this service; further work using an extended follow-up period may be proposed.



For peer review only - http://bmjopen.bmj.com/site/about/guidelines.xhtml

## INTRODUCTION

Common mental disorders (CMDs) including depression, anxiety and alcohol use disorders are highly prevalent conditions that impose a significant societal cost and impact on quality of life [1]. Globally, these conditions have been shown to be the leading cause of non-fatal burden of disease [1], [2]. The disabling impact of these conditions is particularly significant in patients suffering from chronic physical conditions [3]. CMD co-morbidity has been shown to: exacerbate major modifiable risk factors for chronic disease; contribute to chronic disease progression; increase the prevalence of preventable complications and disability; result in poor adherence; and ultimately lead to treatment failure [1], [4]. There is a plethora of evidence on the impact of depression and alcohol use disorders (AUD) on diabetes outcomes [5], [6]. Likewise, alcohol consumption and depressive conditions have been associated with poor HIV outcomes [7], [8]. In addition high-income country (HIC) evidence suggests that patients with these multi-morbidities have higher health care costs even after adjusting for the costs of mental health care [9]; suggesting potential cost savings from investing in mental health treatment for patients with multi-morbidities [10].

There is a pressing need for evidence on cost-effective strategies for integrating mental health care into primary health services, particularly in low-and middle-income countries (LMICs) where health systems are typically fragile and chronically underfunded. In these health systems, an investment in mental health services at primary care level for chronic disease patients has the potential to result in significant returns on investment due to reduced chronic disease treatment costs. South Africa - with the world's largest ART programme [11], an increasing prevalence of diabetes [11] and a huge unmet need for CMD treatment [12] - is one LMIC that may benefit from this integrated approach.

Task-sharing [13], [14] involves re-distributing tasks from specialist providers to non-specialist health workers [15] with specialists providing on-going support to these health workers. Task-sharing is a common strategy for the provision of primary care services in constrained LMIC settings [16]. Due to the long lead times in training mental health staff and financial resource limitations, the task-sharing discourse has found appeal in the policy discussions around introducing and expanding mental health care in LMICs [1], [17]. These discussions are centred on using task-shared approaches [18] to upskill lay health workers to integrate mental health services such as brief counselling interventions into primary care [19], [20].Task-sharing the psychosocial counselling of patients who have chronic co-morbidities to lay health workers is a particularly appealing and potentially cost-effective strategy to treat patients and support their adherence to chronic medication [21], [22] and therefore limit overall health system costs of care [4], [16].

While task-sharing the treatment for CMDs from mental health professionals to lower level health care workers has been suggested as an effective way to deliver services within the context of scarce human resources [4] and has been shown to be cost effective in some HIC settings, there is little if any economic evidence to support the implementation of such strategies in LMICs [4], [16]. This lack of evidence limits decision making around the allocation of resources for these services. Economic evidence on the institutional investment, cost-effectiveness and budget impact of this approach is necessary for empirically-informed health service planning [23]. This work presents a protocol of a cost-effectiveness and cost utility analysis from a societal perspective (i.e. providers and patients) of a task-shared mental health counselling intervention for patients with comorbid HIV or Diabetes in South Africa.



For peer review only - http://bmjopen.bmj.com/site/about/guidelines.xhtml

## METHODS AND ANALYSIS

### Trial design

This study is nested in Project MIND, a cluster randomised control trial across 24 public primary care facilities [24]. Myers et al., (2018) describes the trial in detail [24]. The trial will compare the effectiveness of treatment as usual (MIND_TAU) and two alternative methods for integrating a psychosocial intervention into the primary care services for HIV and Diabetes. Treatment as usual (TAU) is representative of typical primary care level services for CMD in the South African public health service facilities, which is typically limited to referrals [25], [26]. The two models will be the dedicated and designated models of care. In the dedicated approach (MIND_DED), community health workers (CHWs) will be hired and added to the facility staff complement and will dedicate their time to *only* delivering the new counselling service. In the designated approach (MIND_DES) CHWs already working in the facility will be designated to deliver the service *in addition* to their other chronic disease-related activities such as adherence counselling for HIV and health promotion. Supervision and de-briefing of all the counsellors in the active arms will be task-shifted to registered psychological counsellors in line with the national mental health policy framework [27]. Descriptions of the CHWs and registered psychological counsellors roles in the MIND-DED and MIND-DES models and their qualifications and skills levels are detailed in the trial protocol [24]. The Western Cape Department of Health (WCDoH) (WC2016_RP6_9), the South African Medical Research Council (EC 004-2/2015), the University of Cape Town (089/2015), and Oxford University (OxTREC 2-17) approved the study.

### *Intervention and comparator*

The counselling programme delivered in MIND-DED and MIND-DES is based on Motivational Interviewing (MI) and Problem Solving Therapy (PST) [28]. It is delivered in three sessions over six weeks with one optional booster session. The MI component provides psychoeducation about depression and hazardous alcohol use in relation to chronic disease, builds readiness for counselling and helps participants develop goals for counselling. The PST component targets maladaptive coping strategies and builds skills and strategies for dealing with life problems. Patients will receive individual face-to-face counselling from CHWs and take-home booklets to support self-learning between sessions. Registered psychological counsellors, supervised by psychologists, will train the counsellors (see [24] for more detail). The underlying theory, content and evidence for the efficacy of the counselling approach for reducing symptoms of depression and hazardous /harmful alcohol use over the 1 year time frame have been described previously [29]–[31]. In them TAU arm, the standard package of care will be provided to patients who are suspected of having mental health problems. In general, patients using the HIV or diabetes service are asked by a nurse or doctor attending to their care about their mental well-being, life stressors, and use of alcohol or other substances. Patients are provided with advice to change their lifestyles. Where the health care provider deems it necessary, patients are referred to a mental health nurse for further assessment or screening. The patient may also be referred to a social worker who may refer them to NGOs who provide counselling and support services [24].

### *Outcome Measures*

The primary outcomes for the trial are measures of psychosocial functioning, specifically changes in self-reported risk of depression as measured by CES-D (Centre for Epidemiological Studies scale on Depression)[32] and self-reported hazardous alcohol use as measured by the AUDIT (Alcohol Use Disorder Identification Test) [33]. Secondary outcomes include biological measures aimed at assessing changes in chronic disease treatment response, specifically HbA1c for diabetes and HIV-1 RNA viral loads for HIV. Other secondary outcome measures include self-reported adherence to chronic disease medication and health-related quality of life (HRQoL) measured using the EQ-5D [34]. All outcomes



For peer review only - http://bmjopen.bmj.com/site/about/guidelines.xhtml

are measured longitudinally at baseline, and at 6 and 12 month follow-up assessments, recruitment on the trial started in 2017 and will end in February 2019, and final outcome assessments will end in 2020 (see **Figure 1)**.

***Sample Size and Patient Population***

Recruitment will be restricted to consenting adult patients who meet criteria for hazardous alcohol use or depression using the AUDIT or CES-D. These patients are already diagnosed and receiving treatment for diabetes or HIV, but not currently receiving treatment for a CMD. The trial requires a sample size of 1200 patients (600 with HIV and 600 with diabetes) to detect reductions in hazardous alcohol use and risk of depression at 12 month follow-up. The sample size calculations are based on a cluster randomised design with two active arms and a control arm. This clinical trial is powered to detect clinical outcomes, specifically reductions hazardous/ harmful alcohol use and risk of depression at 12-month follow-up rather than economic outcomes [24], [35].

**MIND Economic Evaluation**

**Objective**

The study will be a prospective economic evaluation. The objectives include estimating:

i. Full economic provider costs: of the mental health intervention; any cost offsets attributable to the intervention related to changes to the costs of HIV or Diabetes care at the primary care level; and changes to the costs of referral care (including tuberculosis, emergency department and inpatient care);
ii. Patient costs: associated with the intervention which will include direct OOP payments to private health providers (consultations and medications etc.), travel and subsistence costs and productivity losses.
iii. Cost-effectiveness in terms of the incremental cost per unit of improvement in disease specific outcome (CES-D, AUDIT, HIV-1 RNA, HBA1c);
iv. Cost-utility in terms of the incremental cost per Quality Adjusted Life Year (QALY) gained and;
v. Budget impact of scaling up the intervention.

To achieve these objectives, the incremental cost-effectiveness ratio (ICER) will be calculated by ordering the MIND_DED, the MIND_DES and MIND_TAU alternatives from least cost to highest cost and calculating the difference in costs and outcomes against the next less costly, non-dominated alternative or against the common baseline of MIND_TAU.

**Perspective**

The aim of the economic evaluation is to inform health service decision makers about the costs, outcomes, economic efficiencies and budget impact of introducing task-shared psychosocial counselling to their chronic disease patient body in a resource-scarce context. As such, the economic evaluation will be conducted from a provider perspective, reflecting health service budget constraints. In line with good practise recommendations, the analyses will also be presented from a societal perspective (including both provider and patient perspectives). While public sector primary health care is free at the point of use, patients incur time and travel costs when accessing care and may experience productivity losses; in addition, they may incur costs when using private sector health services. These costs will be collected to inform the patient perspective within the economic evaluation.

**Time Horizon and Discount Rate**

As the health consequences of the intervention will be experienced over a period extending longer than one year, a discount rate will be applied to reflect differentials in timing [36]. A three percent discount rate will be used for discounting both costs and outcomes to allow comparability with other cost-effectiveness analyses [37].



For peer review only - http://bmjopen.bmj.com/site/about/guidelines.xhtml

**Estimating intervention effects**

Single study-based estimates of clinical effectiveness will be obtained from the trial, which will provide the primary and secondary outcome measures previously described. In addition to the disease-specific primary outcomes, and the secondary HIV and diabetes outcomes which will be applied in the cost-effectiveness analysis, the QALY will also be calculated as an end-point that will be used in the cost-utility analysis. The ICER resulting when using a QALY measure represents the additional cost associated with accruing another QALY as one compares strategies (that is, the incremental cost per QALY gained). The QALY allows comparison of ICERs across different diseases and interventions [37]. QALYs are calculated by multiplying the length of time in a health state by the HRQoL of the health state. The EQ-5D is the most widely used instrument for measuring HRQoL for cost-utility analyses [34]. In South Africa it has been validated and translated into Afrikaans and isiXhosa, two of the country's official languages [38]. The EQ-5D is a self-assessment of health status across five domains which include mobility, self-care, usual activities, pain and discomfort and anxiety/depression[39]. In this study, the EQ-5D-3L [34] will be used to measure HRQoL. A single summary index score will be generated using preference weights derived from a time trade-off (TTO) based valuation survey conducted in Zimbabwe in addition to the commonly used United Kingdom value set; the TTO is the most common valuation technique used in LMICs [40]. As the EQ-5D will be administered at all the assessment intervals (baseline, 6 months and 12-months post-enrolment) there will be three observations of the EQ-5D scores for each patient in the trial. The QALY gained per patient over the trial period will be calculated by measuring the area under the quality of life curve [37].

**Estimating costs for the provider's perspective**

Within economic evaluation, the appropriate scope of provider costs includes all costs incurred within the intervention and any changes in broader health system costs that can be attributable to the intervention. We have categorized these costs as intervention costs, HIV and Diabetes service costs and other related provider costs. As shown in **Table 1**, our approach to estimating these costs entails the measurement of quantities of resources that are utilized, and multiplying these quantities by the value (or unit cost) of each resource. These separate steps of measurement and valuation are described below.

| | **MEASUREMENT** | | **VALUATION** | |
|---|---|---|---|---|
| **Cost component** | **Resources used** | **Quantities or utilisation** | **Valuation data** | **Allocation factor** |
| ***INTERVENTION*** | | | | |
| *Capital costs* | | | | |
| Facility Readiness Workshops | Number of staff | Time from trial data | Cost of Employment converted to annual equivalent cost | DED/DES/TAU headcount |
| Intervention training for counsellors | Number of counsellors | Time from trial data | Cost of Employment converted to annual equivalent cost | DED/DES/TAU headcount |
| Counselling room | Space | Square meters | Replacement value converted to annual equivalent cost | DED/DES/TAU headcount |
| Furniture & equipment | e.g. Tape recorders | Inventory | Replacement value converted to annual equivalent cost | DED/DES/TAU headcount |



For peer review only - http://bmjopen.bmj.com/site/about/guidelines.xhtml

| | | | | |
|---|---|---|---|---|
| Vehicles | Vehicles | Inventory | Replacement value converted to annual equivalent cost | DED/DES/TAU headcount |
| *Recurrent costs* | | | | |
| Counselling personnel | Number of staff (Lay counsellors; registered counsellors; clinical psychologist) | Time from time and motion tool | Cost of Employment X proportion of time | DED/DES/TAU headcount |
| Counselling supplies | Manuals, notebooks, pens | Number used - from trial data | Market value | DED/DES/TAU headcount |
| Non-intervention personnel | Number of staff | Time from facility data | Cost of Employment X proportion of time | Facility headcount |
| Utilities | Electricity, water, other utilities, phone, cleaning, transport, and stationery | Facility utilisation | Annual facility expenditure | Facility headcount |
| ***HIV/DIABETES SERVICE*** | | | | |
| *Capital costs* | | | | |
| Buildings | Space used for HIV/Diabetes service | Square meters | Replacement value converted to annual equivalent cost | HIV/Diabetes headcount |
| Equipment& furniture | Equipment and furniture used for HIV/Diabetes service | Inventory | Replacement value converted to annual equivalent cost | HIV/Diabetes headcount |
| Vehicles | Vehicles used for HIV/Diabetes service | Inventory | Replacement value converted to annual equivalent cost | HIV/Diabetes headcount |
| *Recurrent costs* | | | | |
| HIV/Diabetes personnel | Number of staff and staff time spent on HIV/Diabetes service | Time from facility data | Cost of Employment X proportion of time | HIV/Diabetes headcount |
| Drugs | ARV/Diabetes medication | Treatment protocol | National tender prices | Direct allocation |
| Diagnostic tests | CD4 test, diabetes monitoring tests etc. | Treatment protocol | National Health Laboratory Service (NHLS) prices | Direct allocation |
| Non-HIV/Diabetes personnel | Managerial, cleaning and security staff | Facility utilisation | Facility expenditure | Facility headcount |
| Building operating and maintenance | Electricity, water, other utilities, phone, cleaning, transport, and stationery | Facility utilisation | Facility expenditure | Facility headcount |
| ***OTHER RELATED HEALTH CARE PROVIDERS*** | | | | |
| Public clinic visit | Average cost per visit | Patient interviews | Facility expenditure | Facility headcount |
| Public hospital ER/OPD visit | Average cost per OPD visit | Patient Interviews | Facility expenditure | Facility IP days and OP headcount |
| Public hospital Inpatient day | Average cost per inpatient day | Patient Interviews | Facility expenditure | Facility IP days and OP headcount |

**Table 1:** Measuring and valuing provider costs



For peer review only - http://bmjopen.bmj.com/site/about/guidelines.xhtml

***Measurement of resource use***

For the intervention costs, an ingredients approach will be used to estimate resources. Routine data linked to intervention protocols will be collected and used to assess resources directly consumed in the provision of the intervention, including supplies, manuals, and patient education materials. Facility observations of intervention delivery will be conducted for the economist to understand the intervention and the facility context; these observations will complement intervention cost data collection. The costs of intervention focused counsellor training and facility-level institutional strengthening and capacity development through organisational readiness workshops [41] will be included in the cost analysis as start-up costs [42] in order to capture the full economic costs of integrating the intervention into the primary care service for chronic disease patients [42].

A time and motion tool (TMT) developed for this evaluation will measure counsellor time usage in delivering the intervention for both active arms in the study. Time tools have been used in analysis of human resource requirements for expanding access to HIV [43] and TB care [44] using lay health workers and have proved useful in highlighting the scope of duties of this cadre. To our knowledge there is limited evidence in the South African context on time costs of CHW-delivered mental health counselling. The TMT will enable an analysis of differences in counsellor time usage in the different intervention modalities, indicating opportunities for scale and scope efficiencies. It is anticipated that the costs associated with the TAU option will be minimal and any referral costs will be captured through patient self-report of services they have used (see next paragraph).

For the HIV and Diabetes services, a step-down methodology will be used to establish resource use. Staff time will be determined through review of facility organograms and interviews with senior managers. In addition, utilisation of HIV and Diabetes medicine and diagnostics will be estimated by applying standard treatment guidelines/protocols for HIV and Diabetes treatment in primary care services to utilisation statistics obtained from facility records (i.e. protocol-based facility utilisation statistics).

Establishing the use of referral services is challenging given that there is no health information system that enables patient pathways through care to be established. Therefore, to establish patient utilisation of health services beyond their HIV and Diabetes care at the primary level, we will administer a patient questionnaire developed for the study at baseline, and at the 6- and 12-month assessments. Using this questionnaire, we will capture health service utilisation for other primary care services (e.g. TB treatment), hospital services, emergency department care, paramedic care and referrals to mental health providers. Patients will be asked to quantify the number of these additional visits, and lengths of stay in hospital. Given concerns with patient recall, we have asked patients to estimate their usage of ambulatory services over the previous month and have used a recall period of 6 months for inpatient services. These data will then be extrapolated to estimate costs over the time horizon.

***Valuation of costs***

Once the utilisation of services has been established, we will estimate the unit cost of each service. This involves sourcing a value for each item (e.g. annual cost of employment multiplied by proportion of time spent), as well as an allocation factor (e.g. annual facility head count) allowing the estimation of a unit cost (cost of employment X proportion of time / headcount). These sources of data and allocation factors are outlined in detail in **Table 1**.

For capital items, costs will be estimated using the replacement value method [37]. In essence, the current replacement value of each items is calculated and annuitized using data on the estimated useful life of the item and an interest rate representing the opportunity cost component for capital, in order to estimate an equivalent annual cost. An estimated useful life of between 5-20 years for



For peer review only - http://bmjopen.bmj.com/site/about/guidelines.xhtml

furniture and buildings will be applied as while an interest rate of 8% will be used as the annuitization factor based on the rate of return on government bonds in South Africa [37].

**Estimating costs for the patient perspective**

***Measurement of resource use***

For the patient perspective, we will use a questionnaire at baseline, 6 and 12 months to estimate resource use. This will capture any user fees associated with public, private or NGO-provided health services as well as traditional and faith-based therapies. In addition, we will ask patients to estimate their time spent accessing health care services, as well as out of pocket payments for transport, subsistence and accommodation. Travel, subsistence and accommodation costs will relate to both the patient and their caregiver, if the patient is accompanied by a caregiver to any of these services.

***Valuation of costs***

Travel, subsistence and accommodation costs will be estimated using the out of pocket payments reported by patients. Time spent receiving health care will be valued using the equality of wages approach [45], [46]. We will apply this method as public health services in South Africa are typically accessed by those who have a lower socio-economic status. Using this technique, the time costs for all patients are valued at the average income reported by the survey population [45]. This approach is expected to provide a relatively accurate reflection of the opportunity cost of unpaid labour and is favoured over the minimum wage approach in this context as a large proportion of the patients are unemployed resulting in a possible overestimate of the value of the time loss if the minimum wage is used [45]. In addition, we will ask patients if they have lost any income through seeking health care.

**Figure 2** summarises the intervention arms, costs, and outcomes that will be included in this study. All costs will be valued at 2017/2018 prices and, to allow comparability with other studies will be converted from South African Rand (ZAR) to United States Dollars (US$), using appropriate conversion rates for the same period obtained from OANDA, an online currency exchange rate conversion site. Costs incurred during earlier periods (such as manual development) will be inflated using the Consumer Price Index to reflect 2017/2018 prices.

For peer review only



For peer review only - http://bmjopen.bmj.com/site/about/guidelines.xhtml

## Analyses

### *Statistical analysis of costs and effects*

Mean provider costs and patient costs, will be calculated from baseline until the end of the intervention period. In addition, we will explore the extrapolation of costs to later periods if data allow for this. Cost differences between the MIND-TAU, and the MIND-DES and MIND-DED alternatives will be calculated. Sampling uncertainty for cost data will be estimated using statistical methods that account for the non-normal distribution of costs. Effect in terms of CES-D, AUDIT, HIV viral load, HBA1c, and the QALY will be analysed using linear regression on intervention type.

### *Determining cost-effectiveness*

The analysis will be conducted in line with the CHEERS (Consolidated Health Economic Evaluation Reporting Standards) guidelines [47]. Decision analytical models [37] integrating the cost and outcomes of the different intervention alternatives will be developed to assess cost-effectiveness. As a meaningful impact of the MIND-DES and MIND-DED on both costs and effects is anticipated, ICERs will be estimated in terms of incremental cost per natural unit and/or intermediate outcome for the cost effectiveness analysis (CEA), and incremental cost per QALY gained for the cost utility analysis (CUA). As the trial is focused on health systems strengthening for the delivery of mental health services, the decision rule applies to maximising health within the health care budget constraint. Therefore ICERs will be calculated from the provider perspective and , the cost per QALY gained will be compared to the cost-effectiveness thresholds (CETs) for LMIC settings [48]. If our intervention's ICER is less than the chosen CET this will mean that diverting resources to the intervention will increase population health, and if the ICER is more than the CET the intervention is not cost effective. Patient costs will be collected and reported on separately. In addition, to satisfy the requirements of common guidelines [47] and to allow for some degree of comparability with other studies, the ICERs will also be presented for the societal perspective, i.e. including both provider and patient costs.

### *Sensitivity analyses*

The decision analytical models will allow for univariate and multivariate sensitivity analyses to be conducted. This will enable the robustness of study findings to be tested by varying key parameters such as utilisation rates and assessing the impact on cost-effectiveness results. Standard areas of analytical focus will be considered, including testing sensitivity around the resource cost and prices, and theoretical controversies [49] for example: TTO weights [50] and discounting of outcomes [36]. Further analyses to inform the economics of task-sharing will include sensitivity analysis focused on economies and diseconomies of scale and scope, using detailed data from the TMT [51]. This will include testing variations in staff hours worked, variations in staff mix, lay counsellor funding models, and infrastructure investments. The impact of intervention dosage on outcomes will also be assessed in the sensitivity analyses. Statistical methods including multiple imputation will be used to manage the effect of missing information on costs and effects [52]. These sensitivity analyses are summarised in **Table 2**. Probabilistic sensitivity analysis (which allows application of confidence intervals to point estimates) will be used for sensitivity analyses of all relevant individual level variables. The sensitivity analyses will be used to produce a cost-effectiveness acceptability curve (CEAC).

| **Simple sensitivity analysis** | **Base-Case** | **Range** |
|---|---|---|
| Discount rate | 3% | 0%-10% |
| QALYs: HRQoL weights | EQ-5D valued using UK TTO | EQ-5D valued using Zimbabwe TTO |
| Unit costs | Primary data and data from published sources and official statistics for South Africa | Varied within plausible ranges as determined from a literature review of South African cost studies |
| Missing data | Multiple imputation | Complete case analysis |
| **Probabilistic sensitivity analysis** | | |



For peer review only - http://bmjopen.bmj.com/site/about/guidelines.xhtml

| | | |
|---|---|---|
| Intervention utilization data | Mean value | 95% uncertainty interval |
| Referral service utilization data | Mean value | 95% uncertainty interval |
| EQ-5D/CESD/AUDIT data | Mean value | 95% uncertainty interval |
| Clinical outcomes (HbA1c, HIV-1 RNA) | Mean value | 95% uncertainty interval |

**Table 2:** Planned Sensitivity Analyses

***Budget Impact Analysis***

There is an increased demand from decision makers in both LMIC and HIC settings for estimates of the real fiscal implications of investing in health services within a defined budget and budgetary period [53], [54]. Trial based estimates of intervention uptake and population based estimates of number in need of the intervention will be used in budget impact approximations [55] which will be conducted using a mathematical programming approach [56], [57]. Mathematical programming provides a useful equity lens for informing policy decisions around the provision of care in high burden, high cost contexts [58]. It will involve analysing alternative implementation strategies with differing equity implications subject to a budget constraint. This approach aims to support policy making by applying principles of constrained maximisation in resource allocation [58]. The estimates from this study will inform policy-level discussions on the fiscal implications of investing in primary care mental health services.

## ETHICS AND DISSEMINATION

In the CEA we foresee minimal risks to participants, as patient level data will be collected through the main trial, where, in order to minimise the risk of improper disclosure of information study staff will be required to sign a staff confidentiality agreement and will be trained and certified in protecting human participants. Data collection for the trial will be conducted using CAPI in which only a unique study identified is used. Knowledge translation will be through policy briefs, social media and as well as peer-reviewed papers, and presentations at health economics and mental health policy conferences. Engagement with department of health policy makers and other key stakeholders will be through a policy dialogue workshop linked to the main trial's Stake Holder Advisory Group (SAG). Dissemination of results to the public will be through press briefings in national and local media.

## DISCUSSION

The delivery of psychotherapy using varying models of task-sharing to less specialised health workers in primary care has been shown to be cost-effective in HIC settings across a range of mental health conditions [59]–[61]but has limited empirical economic evidence in LMICs [51], [62], [63]. This study will provide evidence to address this gap. Through the cost-effectiveness analysis, the relative efficiency of each delivery mechanism will be established, enabling a discussion around which model produces the best outcomes given the available resources. As staff time is expected to be one of the main drivers of costs due to the nature of the intervention, we have developed a time tool that will allow us to directly measure staff time usage and investigate any potential excess capacity (hypothesized to occur with the MIND-DED counsellors), and scope economies (hypothesized to occur with the MIND-DES counsellors) which would impact the comparative efficiency of the different alternatives and thus the cost effectiveness results, as suggested in a similar study [51]. The cost-utility analysis will aid health service planners in their decision making around resource allocation and may be useful for guiding priority setting decisions both within the mental health budget space and across competing health priorities. To further support policy maker decisions, the affordability of implementing the intervention will need to be assessed, this will require further analysis using trial based evidence on intervention uptake and estimates of population level numbers in need to approximate the budget impact of equitably implementing the service [58].



For peer review only - http://bmjopen.bmj.com/site/about/guidelines.xhtml

Policy discussions on the integration of services to provide holistic care for patients requires economic evidence to support implementation in resource-limited contexts [4], [23]. By estimating the full economic costs of integrating the mental health treatment into the primary care offering for patients with chronic physical diseases, this study will provide much needed economic evidence to support this policy narrative [4], [64], [65]. Empirical data on the costs of upskilling and training staff to deliver the intervention and the time costs of the alternative human resourcing strategies will also aid in understanding how this cadre of staff organise their work and may highlight opportunities for efficiency gains. This is timely in the current policy window where the idea of a “facility counsellor” is being discussed, whose role may span a range of counselling duties including: adherence counselling for TB/HIV, chronic non-communicable physical diseases and mental health counselling. The integration of services is a key health systems focus [66] requiring economic evidence [23]. The integration of services at the primary care level [67] to manage communicable and non-communicable chronicity is a global challenge [20], [22], [68], [69] and a key strategic aim of the Department of Health [70] for expanding access to mental health services and managing chronic diseases in South Africa [27], [70]. Brief interventions for depression and alcohol use disorders lack a common outcome measure for use in cost-effectiveness analysis [71] thus limiting comparison across interventions [72]. This study will provide unique cost-effectiveness evidence using the QALY as an outcome measure for such interventions in the South African context [51]. This will allow comparability of cost effectiveness results [73] and inform value for money decision making [48], [74]. This is of particular importance for South Africa as discussions around cost-effectiveness thresholds [74] are being increasingly presented in the health policy space in the prelude to National Health Insurance. This protocol also responds to the call to publish health economics protocols alongside trial protocols to reduce publication bias of RCT results [75].

Potential challenges to this study include recall bias due to the reliance on patient-reported health service use for our estimations of service utilisation. To limit recall bias, a one month recall period will be used for ambulatory services and 6 month recall for hospitalisations [52]. Another possible limitation is that only a few patients may be referred for mental health care for CMD in the TAU option so we may not fully capture referral costs thus potentially underestimating cost-effectiveness. Another potential limitation is that the short follow-up period of 12 months may not capture the extent of the benefit that patients and the health system could gain through the availability of this service. For example, the degree to which the socio-economic benefits and the returns to mental health associated with such interventions [76] may not be fully evident by the 12 month end-point. This is a common problem associated with trial-based economic evaluations [77].

## FUTURE WORK

Further work associated with this economic evaluation includes the use of modelling techniques to estimate the long-term benefits of the intervention both at the patient and system level and applying cost-benefit techniques to strengthen the evidence base for the mental health investment case.

## CONCLUSION

Priority setting and decisions around the scale up of an integrated comprehensive primary care service need to be informed by micro- and macro-level economic analyses of the investment requirements, impact, and equity implications of delivering key services such as counselling. It is anticipated that the cost estimates, the CEA and budget impact analysis of this intervention will be a useful contribution to priority setting and policy making during the current reconfiguration of South Africa’s health system [78]. It is hoped that the economic evaluation of project MIND will provide much needed evidence on the cost-effectiveness of integrating mental health services into primary care that could support a business case for investing in these services.



For peer review only - http://bmjopen.bmj.com/site/about/guidelines.xhtml

## PATIENT AND PUBLIC INVOLVEMENT

Patients and the public were engaged with through the trial, and were not directly involved in this study

## FUNDING STATEMENT

This work is supported by the joint funded initiatives of the British Medical Research Council, Wellcome Trust and DFID (MR/M014290/1). BM is supported by the South African Medical Research Council.

## COMPETING INTERESTS STATEMENT

The authors declare that they have no competing interests.

## AUTHOR CONTRIBUTIONS

VM-M, SC and BM made substantial contributions to the conception and design of the study. The first draft of the manuscript was written by VM-M and critically revised by all authors (VM-M, BM, KS, CL, TN, SC) for important intellectual content. All authors read and approved the final manuscript.

## ABBREVIATIONS

ART: Antiretroviral therapy; AUDIT: Alcohol Use Disorders Identification Test; CEA: Cost Effectiveness Analysis; CES-D: Center for Epidemiology Scale on Depression; CET: Cost Effectiveness Threshold; CHW: Community health worker; CMD: Common mental disorder; CUA: Cost Utility Analysis; HIC: High Income Country; HRQoL: Health-related quality of life; LMIC: Low- and middle-income country; MI: Motivational interviewing; NCD: Non-communicable disease; PHC: Primary health care; PST: Problem-solving therapy; QALY: Quality-adjusted life year; SAG: Stakeholder Advisory Group; TMT: Time and motion tool; TAU: Treatment as usual; WCDOH: Western Cape Department of Health

## ACKNOWLEDGEMENTS

We thank all WCDOH facilities, which have agreed to participate in the trial, and our Stakeholder Advisory Group for their contributions.

For peer review only



For peer review only - http://bmjopen.bmj.com/site/about/guidelines.xhtml

**REFERENCES**

[1] V. Patel *et al.*, "The Lancet Commission on global mental health and sustainable development," *Lancet*, vol. 392, no. 10157, pp. 1553–1598, 2018.

[2] H. A. Whiteford *et al.*, "Global burden of disease attributable to mental and substance use disorders: findings from the Global Burden of Disease Study 2010," *Lancet*, vol. 382, no. 9904, pp. 1575–1586, 2013.

[3] S. Moussavi, S. Chatterji, E. Verdes, A. Tandon, V. Patel, and B. Ustun, "Depression , chronic diseases , and decrements in health : results from the World Health Surveys," *Lancet*, pp. 851–858, 2007.

[4] V. K. Ngo *et al.*, "Grand Challenges: Integrating Mental Health Care into the Non-Communicable Disease Agenda," *PLoS Med.*, vol. 10, no. 5, pp. 1–6, 2013.

[5] D. Vancampfort *et al.*, "The prevalence of diabetes mellitus type 2 in people with alcohol use disorders: a systematic review and large scale meta-analysis," *Psychiatry Res.*, vol. 246, no. October, pp. 394–400, 2016.

[6] K. Sorsdahl, R. Sewpaul, M. Evans, P. Naidoo, B. Myers, and D. J. Stein, "The association between psychological distress, alcohol use and physical non-communicable diseases in a nationally representative sample of South Africans," *J. Health Psychol.*, vol. 23, no. 4, pp. 618–628, 2018.

[7] M. G. Neuman, M. Schneider, R. M. Nanau, and C. Parry, "Alcohol consumption, progression of disease and other comorbidities, and responses to antiretroviral medication in people living with HIV," *AIDS Res. Treat.*, vol. 2012, 2012.

[8] C. Bernard, F. Dabis, and N. De Rekeneire, "Prevalence and factors associated with depression in people living with HIV in sub-Saharan Africa: A systematic review and meta-analysis," *PLoS One*, vol. 12, no. 8, pp. 1–22, 2017.

[9] L. E. Egede, D. Zheng, and K. Simpson, "Comorbid depression is associated with increased health care use and expenditures in individuals with diabetes," *Diabetes Care*, vol. 25, no. 3, pp. 464–470, 2002.

[10] W. J. Katon, J. E. Russo, M. Von Korff, E. H. B. Lin, E. Ludman, and P. S. Ciechanowski, "Long-term effects on medical costs of improving depression outcomes in patients with depression and diabetes," *Diabetes Care*, vol. 31, no. 6, pp. 1155–1159, 2008.

[11] B. M. Mayosi, J. E. Lawn, A. van Niekerk, D. Bradshaw, S. S. Abdool Karim, and H. M. Coovadia, "Health in South Africa: changes and challenges since 2009.," *Lancet*, vol. 380, no. 9858, pp. 2029–43, Dec. 2012.

[12] D. J. Stein *et al.*, "Lifetime prevalence of psychiatric disorders in South Africa.," *Br. J. Psychiatry*, vol. 192, pp. 112–117, 2008.

[13] B. D. Fulton, R. M. Scheffler, S. P. Sparkes, E. Y. Auh, M. Vujicic, and A. Soucat, "Health workforce skill mix and task shifting in low income countries: a review of recent evidence.," *Hum. Resour. Health*, vol. 9, no. 1, p. 1, 2011.

[14] WHO, "Treat, Train, Retain: Task Shifting Global Recomendations and Guidelines," *World Heal. Organ.*, p. 96, 2008.

[15] WHO, "mhGAP Intervention Guide for mental, neurological and substance use disorders in non-specialized health settings Version 2.0," *mhGAP Newsl.*, vol. 1, no. 9, pp. 1–4, 2015.

[16] R. Joshi *et al.*, "Task Shifting for Non-Communicable Disease Management in Low and Middle



For peer review only - http://bmjopen.bmj.com/site/about/guidelines.xhtml

Income Countries – A Systematic Review," *PLoS One*, vol. 9, no. 8, p. e103754, 2014.

[17] D. Javadi, I. Feldhaus, A. Mancuso, and A. Ghaffar, "Applying systems thinking to task shifting for mental health using lay providers: a review of the evidence," *Glob. Ment. Heal.*, vol. 4, p. e14, 2017.

[18] T. A. Bruckner *et al.*, "The mental health workforce gap in low- and middle-income countries: A needs-based approach," *Bull. World Health Organ.*, vol. 89, no. 3, pp. 184–194, 2011.

[19] J. Mugisha, M. De Hert, B. Stubbs, D. Basangwa, and D. Vancampfort, "Physical health policies and metabolic screening in mental health care systems of Sub Saharan african Countries: a s systematic review," *Int. J. Ment. Health Syst.*, pp. 1–7, 2017.

[20] G. Thornicroft *et al.*, "Integrated care for people with long-term mental and physical health conditions in low-income and middle-income countries," *The Lancet Psychiatry*, vol. 0366, no. 18, pp. 1–13, 2018.

[21] J. E. Haberer *et al.*, "Improving antiretroviral therapy adherence in resource-limited settings at scale: A discussion of interventions and recommendations," *J. Int. AIDS Soc.*, vol. 20, no. 1, pp. 1–15, 2017.

[22] F. L. H. Chuah *et al.*, "Interventions and approaches to integrating HIV and mental health services: a systematic review," *Health Policy Plan.*, 2017.

[23] S. I. Watson, H. Sahota, C. A. Taylor, Y.-F. Chen, and R. J. Lilford, "Cost-effectiveness of health care service delivery interventions in low and middle income countries: a systematic review," *Glob. Heal. Res. Policy*, vol. 3, no. 17, 2018.

[24] B. Myers *et al.*, "Comparing dedicated and designated models of integrating mental health into chronic disease care : study protocol for a cluster randomized controlled trial," *Trials*, vol. 19, no. 185, pp. 1–13, 2018.

[25] C. Lund, S. Kleintjes, R. Kakuma, and A. J. Flisher, "Public sector mental health systems in South Africa: Inter-provincial comparisons and policy implications," *Soc. Psychiatry Psychiatr. Epidemiol.*, vol. 45, no. 3, pp. 393–404, 2010.

[26] I. Petersen and C. Lund, "Mental health service delivery in South Africa from 2000 to 2010: One step forward, one step back," *South African Med. J.*, vol. 101, no. 10, pp. 751–757, 2011.

[27] National Department of Health Republic of South Africa, "National Mental Health Policy Framework and Strategic Plan 2013-2020," Pretoria, 2013.

[28] K. Sorsdahl *et al.*, "Adapting a blended motivational interviewing and problem-solving intervention to address risky substance use amongst South Africans.," *Psychother. Res.*, vol. 3307, no. April, pp. 37–41, 2014.

[29] B. Myers, D. J. Stein, B. Mtukushe, and K. Sorsdahl, "Feasibility and Acceptability of Screening and Brief Interventions to Address Alcohol and Other Drug Use among Patients Presenting for Emergency Services in Cape Town, South Africa.," *Adv. Prev. Med.*, vol. 2012, p. 569153, 2012.

[30] K. Sorsdahl *et al.*, "Screening and brief interventions for substance use in emergency departments in the Western Cape province of South Africa: Views of health care professionals," *Int. J. Inj. Contr. Saf. Promot.*, vol. 21, no. 3, pp. 236–243, 2014.

[31] K. Sorsdahl *et al.*, "The efficacy of a blended motivational interviewing and problem solving therapy intervention to reduce substance use among patients presenting for emergency services in South Africa: A randomized controlled trial," *Subst. Abus. Treat. Prev. Policy*, vol.



For peer review only - http://bmjopen.bmj.com/site/about/guidelines.xhtml

10, no. 1, pp. 1–10, 2015.

[32] N. T. Van Dam and M. Earleywine, “Validation of the Center for Epidemiologic Studies Depression Scale-Revised (CESD-R): Pragmatic depression assessment in the general population,” *Psychiatry Res.*, vol. 186, no. 1, pp. 128–132, 2011.

[33] T. F. Babor, J. C. Higgins-Biddle, J. B. Saunders, and M. G. Monteiro, “The Alcohol Use Disorders Identification Test Guidelines for Use in Primary Care,” *World Heal. Organ.*, pp. pp1-40, 2001.

[34] M. van Reenen and B. Janssen, “EQ-5D-5L User Guide - Basic information on how to use the EQ-5D-5L instrument.,” *Version 2.1*, no. October, 2015.

[35] W. Hollingworth, D. McKell-Redwood, L. Hampson, and C. Metcalfe, “Cost-utility analysis conducted alongside randomized controlled trials: Are economic end points considered in sample size calculations and does it matter?,” *Clin. Trials*, vol. 10, no. 1, pp. 43–53, 2013.

[36] A. E. Attema, W. B. F. Brouwer, and K. Claxton, “Discounting in Economic Evaluations,” *Pharmacoeconomics*, vol. 36, no. 7, pp. 745–758, 2018.

[37] M. F. Drummond, M. J. Sculpher, K. Claxton, G. L. Stoddart, and G. W. Torrance, *Methods for the Economic Evaluation of Health Care Programmmes*, 4th ed. Oxford: Oxford University Press, 2015.

[38] J. Jelsma, S. Mkoka, L. Amosun, and J. Nieuwveldt, “The reliability and validity of the Xhosa version of the EQ-5D.,” *Disabil. Rehabil.*, vol. 26, no. 2, pp. 103–108, 2004.

[39] R. Rabin and F. De Charro, “EQ-5D: A measure of health status from the EuroQol Group,” *Ann. Med.*, vol. 33, no. 5, pp. 337–343, 2001.

[40] S. Kularatna, J. A. Whitty, N. W. Johnson, and P. A. Scuffham, “Health State Valuation in Low- and Middle-Income Countries: A Systematic Review of the Literature,” *Value Heal.*, vol. 16, no. 6, pp. 1091–1099, 2013.

[41] C. Brooke-Sumner, K. Sorsdahl, C. Lombard, P. Petersen-Williams, and B. Myers, “Protocol for development and validation of a context-appropriate tool for assessing organisational readiness for change in primary health clinics in South Africa,” *BMJ Open*, vol. 8, no. 4, p. e020539, 2018.

[42] A. Vassall *et al.*, “Reference Case for Estimating the Costs of Global Health Services and Interventions,” Global Health Cost Consortium, 2017.

[43] H. Naburi *et al.*, “The potential of task-shifting in scaling up services for prevention of mother-to-child transmission of HIV: a time and motion study in Dar es Salaam, Tanzania.,” *Hum. Resour. Health*, vol. 15, no. 1, p. 35, 2017.

[44] W. A. Odendaal and S. Lewin, “The provision of TB and HIV/AIDS treatment support by lay health workers in South Africa: a time-and-motion study.,” *Hum. Resour. Health*, vol. 12, no. 1, p. 18, 2014.

[45] N. Foster, A. Vassall, S. Cleary, L. Cunnama, G. Churchyard, and E. Sinanovic, “The economic burden of TB diagnosis and treatment in South Africa,” *Soc. Sci. Med.*, vol. 130, pp. 42–50, 2015.

[46] V. Mauch, N. Woods, B. Kirubi, H. Kipruto, J. Sitienei, and E. Klinkenberg, “Assessing access barriers to tuberculosis care with the tool to Estimate Patients’ Costs: Pilot results from two districts in Kenya,” *BMC Public Health*, vol. 11, pp. 18–21, 2011.



For peer review only - http://bmjopen.bmj.com/site/about/guidelines.xhtml

[47] D. Husereau *et al.*, “Consolidated Health Economic Evaluation Reporting Standards (CHEERS) statement,” *Eur. J. Heal. Econ.*, vol. 14, no. 3, pp. 367–372, 2013.

[48] B. Woods, P. Revill, M. Sculpher, and K. Claxton, “Country-Level Cost-Effectiveness Thresholds: Initial Estimates and the Need for Further Research,” *Value Heal.*, vol. 19, no. 8, pp. 929–935, 2016.

[49] A. M. Gray and T. Wilkinson, “Economic evaluation of healthcare interventions: Old and new directions,” *Oxford Rev. Econ. Policy*, vol. 32, no. 1, pp. 102–121, 2016.

[50] A. Manca, N. Hawkins, and M. J. Sculpher, “Estimating mean QALYs in trial-based cost-effectiveness analysis: The importance of controlling for baseline utility,” *Health Econ.*, vol. 14, no. 5, pp. 487–496, 2005.

[51] R. Dwommoh *et al.*, “Brief interventions to address substance use among patients presenting to emergency departments in resource poor settings : a cost ‑ effectiveness analysis,” *Cost Eff. Resour. Alloc.*, pp. 1–11, 2018.

[52] D. Hughes *et al.*, “Conducting Economic Evaluations Alongside Randomised Trials: Current Methodological Issues and Novel Approaches,” *Pharmacoeconomics*, vol. 34, no. 5, pp. 447–461, 2016.

[53] N. Carvalho, M. Jit, S. Cox, J. Yoong, and R. C. W. Hutubessy, “Capturing Budget Impact Considerations Within Economic Evaluations: A Systematic Review of Economic Evaluations of Rotavirus Vaccine in Low- and Middle-Income Countries and a Proposed Assessment Framework,” *Pharmacoeconomics*, vol. 36, no. 1, pp. 79–90, 2018.

[54] S. Ghabri and J. Mauskopf, “The use of budget impact analysis in the economic evaluation of new medicines in Australia, England, France and the United States: relationship to cost-effectiveness analysis and methodological challenges,” *Eur. J. Heal. Econ.*, vol. 19, no. 2, pp. 173–175, 2018.

[55] S. D. Sullivan *et al.*, “Budget impact analysis - Principles of good practice: Report of the ISPOR 2012 budget impact analysis good practice II task force,” *Value Heal.*, vol. 17, no. 1, pp. 5–14, 2014.

[56] A. A. Stinnett and A. D. Paltiel, “Allocation of Health Care Resources,” *J. Health Econ.*, vol. 15, 1996.

[57] D. M. Epstein, Z. Chalabi, K. Claxton, and M. Sculpher, “Efficiency, equity, and budgetary policies: Informing decisions using mathematical programming,” *Med. Decis. Mak.*, vol. 27, no. 2, pp. 128–137, 2007.

[58] S. Cleary, G. Mooney, and D. Mcintyre, “Equity and Efficiency in HIV - Treatment in South Africa: The Contribution of Mathematical Programming to Priority Setting.,” *Health Econ.*, vol. 19, pp. 1166–1180, 2010.

[59] W. Katon *et al.*, “Cost-effectiveness of a multicondition collaborative care intervention,” *Arch. Gen. Psychiatry*, vol. 69, no. 5, pp. 506–514, 2012.

[60] J. W. Hay, W. J. Katon, K. Ell, P.-J. Lee, and J. J. Guterman, “Cost-Effectiveness Analysis of Collaborative Care Management of Major Depression among Low-Income, Predominantly Hispanics with Diabetes,” *Value Heal.*, vol. 15, no. 2, pp. 249–254, 2012.

[61] D. A. Richards *et al.*, “Clinical effectiveness of collaborative care for depression in UK primary care (CADET): cluster randomised controlled trial,” *BMJ*, vol. 347, no. 14, p. f4913, 2013.

[62] V. Patel, D. Chisholm, S. Rabe-Hesketh, F. Dias-Saxena, G. Andrew, and A. Mann, “Efficacy and



For peer review only - http://bmjopen.bmj.com/site/about/guidelines.xhtml

cost-effectiveness of drug and psychological treatments for common mental disorders in general health care in Goa, India: a randomised, controlled trial.," *Lancet (London, England)*, vol. 361, no. 9351, pp. 33–9, 2003.

[63] D. Siskind, F. K. Baingana, and J. Kim, "Cost-Effectiveness of Group Psycotherapy Depression For Depression in Uganda," *J. Ment. Heal. Policy Econ.*, vol. 11, pp. 127–133, 2008.

[64] I. Petersen, C. Lund, A. Bhana, and A. J. Flisher, "A task shifting approach to primary mental health care for adults in South Africa: Human resource requirements and costs for rural settings," *Health Policy Plan.*, vol. 27, no. 1, pp. 42–51, 2012.

[65] H. Jack *et al.*, "Closing the mental health treatment gap in South Africa: A review of costs and cost-effectiveness," *Glob. Health Action*, vol. 7, no. SUPP.1, pp. 1–11, 2014.

[66] R. Atun, T. De Jongh, F. Secci, K. Ohiri, and O. Adeyi, "Integration of targeted health interventions into health systems: A conceptual framework for analysis," *Health Policy Plan.*, vol. 25, no. 2, pp. 104–111, 2010.

[67] L. Dudley and P. Garner, "Strategies for integrating primary health services in low- and middle-income countries at the point of delivery.," *Cochrane Database Syst. Rev.*, no. 2, p. CD003318, 2011.

[68] V. Haldane *et al.*, "Integrating cardiovascular diseases, hypertension, and diabetes with HIV services: a systematic review," *AIDS Care - Psychol. Socio-Medical Asp. AIDS/HIV*, vol. 30, no. 1, pp. 103–115, 2018.

[69] C. G. Kemp *et al.*, "Implementation science for integration of HIV and non-communicable disease services in sub-Saharan Africa: A systematic review," *Aids*, vol. 32, no. May, pp. S93–S105, 2018.

[70] Western Cape Government, "Healthcare 2030: The Road to Wellness," Cape Town, 2014.

[71] A. J. Cowell, J. M. Brown, M. J. Mills, R. H. Bender, and B. J. Wedehase, "Cost-effectiveness analysis of motivational interviewing with feedback to reduce drinking among a sample of college students," *J. Stud. Alcohol Drugs*, vol. 73, no. 2, pp. 226–37, 2012.

[72] A. J. Cowell, J. W. Bray, M. J. Mills, and J. M. Hinde, "Conducting economic evaluations of screening and brief intervention for hazardous drinking: Methods and evidence to date for informing policy," *Drug Alcohol Rev.*, vol. 29, no. 6, pp. 623–630, 2010.

[73] M. C. Weinstein, G. Torrance, and A. Mcguire, "QALYs : The Basics," vol. 12, 2009.

[74] P. Thokala, J. Ochalek, A. A. Leech, and T. Tong, "Cost-Effectiveness Thresholds: the Past, the Present and the Future," *Pharmacoeconomics*, pp. 1–14, 2018.

[75] T. Powell-Jackson *et al.*, "Trials and tribulations: cross-learning from the practices of epidemiologists and economists in the evaluation of public health interventions," *Health Policy Plan.*, no. May, pp. 1–5, 2018.

[76] C. Lund *et al.*, "Poverty and common mental disorders in low and middle income countries: A systematic review.," *Soc. Sci. Med.*, vol. 71, no. 3, pp. 517–528, 2010.

[77] H. A. Glick, J. A. Doshi, S. S. Sonnad, and D. Polsky, *Economic Evauation in Clinical Trials*, 2nd ed. New York: Oxford University Press, 2015.

[78] L. Rispel, J. Moorman, M. Chersich, J. Goudge, N. Nxumalo, and T. Ndou, "REVITALISING PRIMARY HEALTH CARE IN SOUTH AFRICA: Review of primary health care package, norms and standards," Johannesburg, 2010.



For peer review only - http://bmjopen.bmj.com/site/about/guidelines.xhtml

**Figure 1** Economic Evaluation: Analyses, Outcomes, Measurement and Assessment Timing

**Figure 2** Project MIND: Intervention Arms, Costs, Outcomes & Economic Evaluation

**Table 1:** Measuring and valuing provider costs

**Table 2:** Planned Sensitivity Analyses

For peer review only



For peer review only - http://bmjopen.bmj.com/site/about/guidelines.xhtml

| Analysis | Assessment/ Economic Evaluation Outcomes | Measurement instrument OR Clinical Parameter | Assessment Timing (Trial recruitment: starts 2017- ends 2019) | | |
|---|---|---|---|---|---|
| | | Intervention start → | Baseline | 6 months | 1 Year ← Intervention assessment end |
| | ***1º Outcomes*** | | | | |
| CEA | Harmful alcohol use (Self-report interview) | AUDIT | ✓ | ✓ | ✓ |
| CEA | Depression (Self-report interview) | CES-D | ✓ | ✓ | ✓ |
| CEA | ***2º Outcomes*** | | | | |
| CEA | Diabetes treatment failure risk | HbA1c | ✓ | ✓ | ✓ |
| CEA | | BMI | | | |
| CEA | HIV treatment failure risk | HIV-1 RNA (HIV-viral load) | ✓ | ✓ | ✓ |
| CEA | Chronic Disease Medication Adherence | Visual analogue Scale and CASE adherence Index | ✓ | ✓ | ✓ |
| CUA | ***Multi-attribute Outcomes*** | | | | |
| CUA | QALY | EQ-5D | ✓ | ✓ | ✓ |

For peer review only

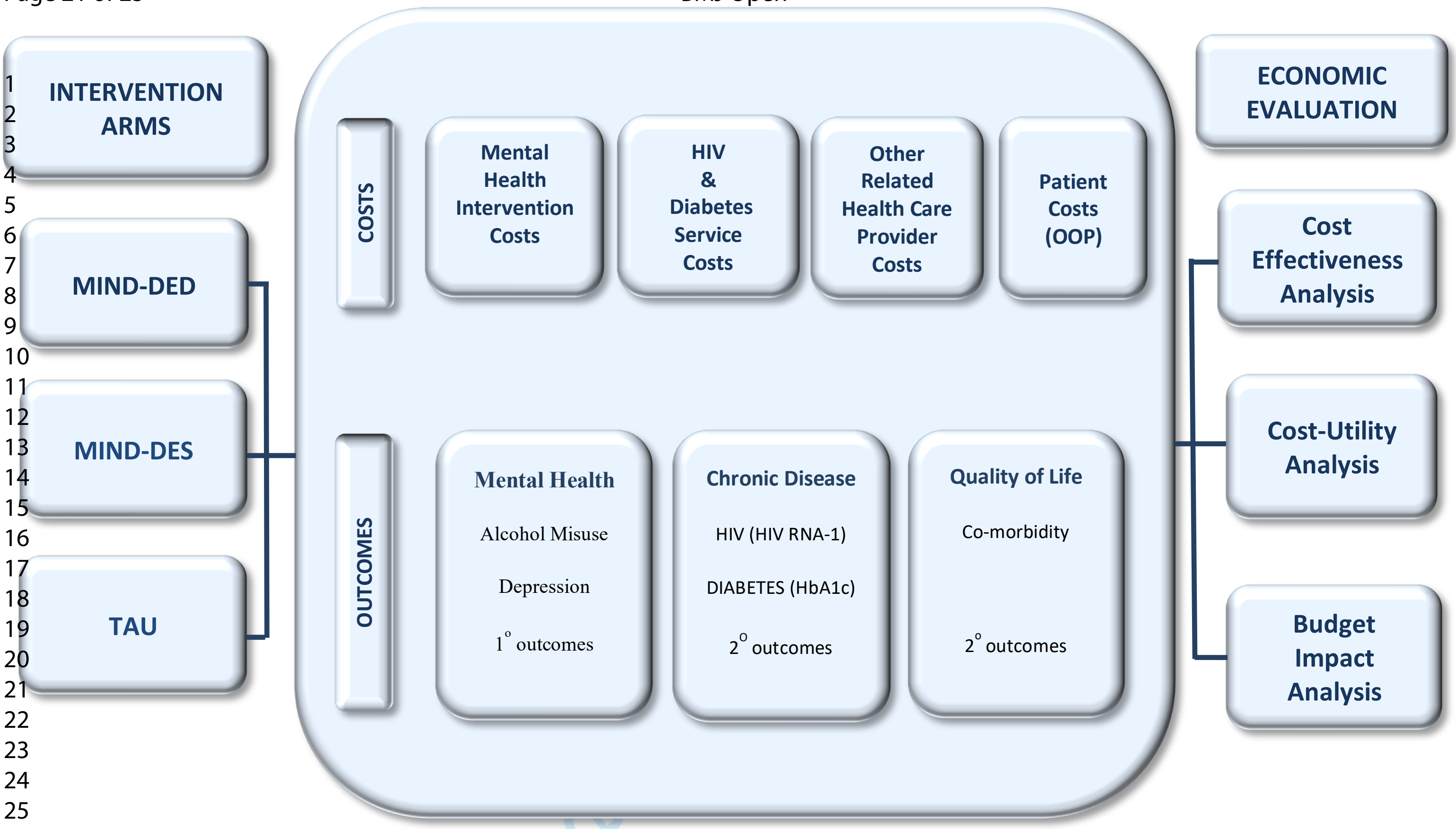
INTERVENTION ARMS
MIND-DED
MIND-DES
TAU
COSTS
Mental Health Intervention Costs
HIV & Diabetes Service Costs
Other Related Health Care Provider Costs
Patient Costs (OOP)
OUTCOMES
Mental Health
Alcohol Misuse
Depression
1° outcomes
Chronic Disease
HIV (HIV RNA-1)
DIABETES (HbA1c)
2° outcomes
Quality of Life
Co-morbidity
2° outcomes
ECONOMIC EVALUATION
Cost Effectiveness Analysis
Cost-Utility Analysis
Budget Impact Analysis

For peer review only

**CHEERS checklist**
**Items to include when reporting economic evaluations of health interventions**

Further discussion of the checklist may be accessed from the ISPOR Health Economic Evaluation Publication Guidelines – CHEERS: Good Reporting Practices webpage: http://www.ispor.org/TaskForces/EconomicPubGuidelines.asp.

| Section /item | Item No | Recommendation | Reported on page No/ line No |
|---|---|---|---|
| **Title and abstract** | | | |
| Title | 1 | Identify the study as an economic evaluation or use more specific terms such as "cost-effectiveness analysis", and describe the interventions compared. | Page 1 |
| Abstract | 2 | Provide a structured summary of objectives, perspective, setting, methods (including study design and inputs), results (including base case and uncertainty analyses), and conclusions. | Page 2 |
| **Introduction** | | | |
| Background and objectives | 3 | Provide an explicit statement of the broader context for the study. | Page 3 |
| | | Present the study question and its relevance for health policy or practice decisions. | Page 5 |
| **Methods** | | | |
| Target population and subgroups | 4 | Describe characteristics of the base case population and subgroups analysed, including why they were chosen. | Page 5 |
| Setting and location | 5 | State relevant aspects of the system(s) in which the decision(s) need(s) to be made. | Page 4 |
| Study perspective | 6 | Describe the perspective of the study and relate this to the costs being evaluated. | Page 5,6,7 |
| Comparators | 7 | Describe the interventions or strategies being compared and state why they were chosen. | Page 4 |
| Time horizon | 8 | State the time horizon(s) over which costs and consequences are being evaluated and say why appropriate. | Page 5 |
| Discount rate | 9 | Report the choice of discount rate(s) used for costs and outcomes and say why appropriate. | Page 5 |
| Choice of health outcomes | 10 | Describe what outcomes were used as the measure(s) of benefit in the evaluation and their relevance for the type of analysis performed. | Page 4 |
| Measurement of effectiveness | 11a | *Single study-based estimates:* Describe fully the design features of the single effectiveness study and why the single study was a sufficient source of clinical effectiveness data. | Page 6 |
| | 11b | *Synthesis-based estimates*: Describe fully the methods used for identification of included studies and synthesis of clinical effectiveness data. | Not applicable |
| Measurement and valuation of preference based outcomes | 12 | If applicable, describe the population and methods used to elicit preferences for outcomes. | Page 6 |
| Estimating resources and costs | 13a | *Single study-based economic evaluation:* Describe approaches used to estimate resource | Page 6,7,8 |

For peer review only

| Section /item | Item No | Recommendation | Reported on page No/ line No |
|---|---|---|---|
| | | use associated with the alternative interventions. Describe primary or secondary research methods for valuing each resource item in terms of its unit cost. Describe any adjustments made to approximate to opportunity costs. | |
| | 13b | *Model-based economic evaluation:* Describe approaches and data sources used to estimate resource use associated with model health states. Describe primary or secondary research methods for valuing each resource item in terms of its unit cost. Describe any adjustments made to approximate to opportunity costs. | Not applicable |
| Currency, price date, and conversion | 14 | Report the dates of the estimated resource quantities and unit costs. Describe methods for adjusting estimated unit costs to the year of reported costs if necessary. Describe methods for converting costs into a common currency base and the exchange rate. | Page 9 |
| Choice of model | 15 | Describe and give reasons for the specific type of decision-analytical model used. Providing a figure to show model structure is strongly recommended. | Protocol – not applicable |
| Assumptions | 16 | Describe all structural or other assumptions underpinning the decision-analytical model. | Protocol – not applicable |
| Analytical methods | 17 | Describe all analytical methods supporting the evaluation. This could include methods for dealing with skewed, missing, or censored data; extrapolation methods; methods for pooling data; approaches to validate or make adjustments (such as half cycle corrections) to a model; and methods for handling population heterogeneity and uncertainty. | Page 10 |
| **Results** | | | |
| Study parameters | 18 | Report the values, ranges, references, and, if used, probability distributions for all parameters. Report reasons or sources for distributions used to represent uncertainty where appropriate. Providing a table to show the input values is strongly recommended. | Protocol – not applicable |
| Incremental costs and outcomes | 19 | For each intervention, report mean values for the main categories of estimated costs and outcomes of interest, as well as mean differences between the comparator groups. If applicable, report incremental cost-effectiveness ratios. | Protocol – not applicable |
| Characterising uncertainty | 20a | *Single study-based economic evaluation:* Describe the effects of sampling uncertainty for the estimated incremental cost and incremental effectiveness parameters, together with the impact of methodological assumptions (such as discount rate, study perspective). | Protocol – not applicable |
| | 20b | *Model-based economic evaluation:* Describe the effects on the results of uncertainty for all input parameters, and uncertainty related to the | Not applicable |

For peer review only

| Section /item | Item No | Recommendation | Reported on page No/ line No |
|---|---|---|---|
| | | structure of the model and assumptions. | |
| Characterising heterogeneity | 21 | If applicable, report differences in costs, outcomes, or cost-effectiveness that can be explained by variations between subgroups of patients with different baseline characteristics or other observed variability in effects that are not reducible by more information. | Protocol – not applicable |
| **Discussion** | | | |
| Study findings, limitations, generalisability, and current knowledge | 22 | Summarise key study findings and describe how they support the conclusions reached. Discuss limitations and the generalisability of the findings and how the findings fit with current knowledge. | Protocol discussion |
| **Other** | | | |
| Source of funding | 23 | Describe how the study was funded and the role of the funder in the identification, design, conduct, and reporting of the analysis. Describe other non-monetary sources of support. | Page 11 |
| Conflicts of interest | 24 | Describe any potential for conflict of interest of study contributors in accordance with journal policy. In the absence of a journal policy, we recommend authors comply with International Committee of Medical Journal Editors recommendations. | Information provided via the submission system |

For consistency, the CHEERS statement checklist format is based on the format of the CONSORT statement checklist

The citation for the CHEERS Task Force Report is: Husereau D, Drummond M, Petrou S, et al. Consolidated health economic evaluation reporting standards (CHEERS)—Explanation and elaboration: A report of the ISPOR health economic evaluations publication guidelines good reporting practices task force. Value Health 2013;16:231-50.

For peer review only
